# Differential Suppression of *Nicotiana benthamiana* Innate Immune Responses by Transiently Expressed *Pseudomonas syringae* Type III Effectors

**DOI:** 10.3389/fpls.2018.00688

**Published:** 2018-05-23

**Authors:** Selena Gimenez-Ibanez, Dagmar R. Hann, Jeff H. Chang, Cécile Segonzac, Thomas Boller, John P. Rathjen

**Affiliations:** ^1^The Sainsbury Laboratory, Norwich, United Kingdom; ^2^Plant Molecular Genetics Department, Centro Nacional de Biotecnología-Consejo Superior de Investigaciones Científicas, Madrid, Spain; ^3^Department of Environmental Sciences, Botanical Institute, University of Basel, Basel, Switzerland; ^4^Institute of Genetics, Ludwig-Maximilians-Universität München, Munich, Germany; ^5^Department of Botany and Plant Pathology, Oregon State University, Corvallis, OR, United States; ^6^Center for Genome Research and Biocomputing, Oregon State University, Corvallis, OR, United States; ^7^Department of Plant Science, Plant Genomics and Breeding Institute and Research Institute of Agriculture and Life Sciences, College of Agriculture and Life Sciences, Seoul National University, Seoul, South Korea; ^8^Research School of Biology, Australian National University, Acton, ACT, Australia

**Keywords:** *Pseudomonas*, effector, PTI, cell death, suppression, *Nicotiana benthamiana*

## Abstract

The plant pathogen *Pseudomonas syringae* injects about 30 different virulence proteins, so-called effectors, via a type III secretion system into plant cells to promote disease. Although some of these effectors are known to suppress either pattern-triggered immunity (PTI) or effector-triggered immunity (ETI), the mode of action of most of them remains unknown. Here, we used transient expression in *Nicotiana benthamiana*, to test the abilities of type III effectors of *Pseudomonas syringae* pv. tomato (*Pto*) DC3000 and *Pseudomonas syringae* pv. *tabaci* (*Pta*) 11528 to interfere with plant immunity. We monitored the sequential and rapid bursts of cytoplasmic Ca^2+^ and reactive oxygen species (ROS), the subsequent induction of defense gene expression, and promotion of cell death. We found that several effector proteins caused cell death, but independently of the known plant immune regulator *NbSGT1*, a gene essential for ETI. Furthermore, many effectors delayed or blocked the cell death-promoting activity of other effectors, thereby potentially contributing to pathogenesis. Secondly, a large number of effectors were able to suppress PAMP-induced defense responses. In the majority of cases, this resulted in suppression of all studied PAMP responses, suggesting that these effectors target common elements of PTI. However, effectors also targeted different steps within defense pathways and could be divided into three major groups based on their suppressive activities. Finally, the abilities of effectors of both *Pto* DC3000 and *Pta* 11528 to suppress plant immunity was conserved in most but not all cases. Overall, our data present a comprehensive picture of the mode of action of these effectors and indicate that most of them suppress plant defenses in various ways.

## Introduction

Plants respond to infections via an innate immune system that employs external and internal receptors (Jones and Dangl, 2006). Primary perception is based on the ability to discriminate between self and non-self. Plants rely on surface-localized pattern recognition receptors (PRRs) to detect conserved molecules called pathogen-associated molecular patterns (PAMPs), or sense wound- and injury-related molecules called danger-associated molecular patterns (DAMPs) to infer the presence of microbes (Boller and Felix, 2009). This recognition leads to PAMP-triggered immunity (PTI), which is considered to be the first level of plant defense, and restricts pathogen infection in most plant species. To overcome such immunity, successful bacterial pathogens use type III secretion systems (TTSSs) to deliver virulence molecules called effector proteins into eukaryotic cells (Collmer et al., 2002). The second branch of the plant innate immune system recognizes type III effectors inside the plant cell via nucleotide-binding leucine-rich repeat (NB-LRR) resistance (R) proteins and is called effector-triggered immunity (ETI) (Chisholm et al., 2006). This specific recognition leads to strong activation of a similar range of plant responses as PTI and is characteristically associated with programmed cell death known as the hypersensitive response (HR) at the infection site.

Pattern recognition receptors are generally membrane-bound receptor kinases (RKs) or receptor proteins (RPs). Ligand perception at the cell surface causes the immediate formation of stable heterodimers comprised of the specific PRR and certain co-receptors. This constitutes an active receptor complex that initiates intracellular signaling (Chinchilla et al., 2007; Heese et al., 2007; Boutrot et al., 2010; Liu et al., 2012; Cao et al., 2014). In *Arabidopsis*, the paradigm for PAMP sensing is the perception of the bacterial flagellin protein by the leucine-rich repeat (LRR) RK FLS2 (FLAGELLIN-SENSING 2), which triggers formation of an active receptor complex with the LRR-RK co-receptor BAK1 (BRI1-ASSOCIATED KINASE 1) (Chinchilla et al., 2007; Heese et al., 2007). Similarly, chitin oligosaccharides released from fungal cell walls are perceived by the *Arabidopsis* lysin motif (LysM) RKs LYK5 (LYSIN MOTIF-CONTAINING RECEPTOR-LIKE KINASE 3) and CERK1 (CHITIN ELICITOR RECEPTOR KINASE 1) that form a receptor complex in which both bind chitin (Liu et al., 2012; Cao et al., 2014). Upon PAMP sensing, the activation of different PRR complexes leads to the selective phosphorylation and activation of specific cytoplasmic receptor-like cytoplasmic kinases (RLCKs). These include BIK1 (BOTRYTIS-INDUCED KINASE 1) downstream of flagellin recognition, and PBL27 downstream of chitin perception (Lu et al., 2010; Kadota et al., 2014; Shinya et al., 2014; Yamada et al., 2016).

Activation of PRRs induces a variety of host responses, including evolution of a burst of Ca^2+^ and reactive oxygen species (ROS), post-translational activation of mitogen-activated protein kinases (MAPK), induction of defense genes, callose deposition into plant cell walls and functional immunity (Kunze et al., 2004; Zipfel et al., 2004; Chinchilla et al., 2006). Upon PAMP stimulation, one of the earliest responses is changes in ion fluxes across the plasma membrane. This includes an influx of Ca^2+^ from the apoplast within seconds (Chandra et al., 1997; Blume et al., 2000; Lecourieux et al., 2002; Davies et al., 2006; Segonzac et al., 2011). Downstream of the Ca^2+^ influx, two distinct branches of signaling occur, one leading to ROS production by NADPH oxidases and the other to the activation of MAPKs and transcriptional changes (Segonzac et al., 2011). The plasma membrane *Arabidopsis* NADPH oxidase RBOHD (RESPIRATORY BURST OXIDASE HOMOLOG D) is part of the PRR complex and is directly regulated through phosphorylation by the RLCK BIK1 in a calcium-independent manner. This leads to ROS production and antibacterial immunity (Kadota et al., 2014; Li et al., 2014). In contrast, the RLCK PBL27 directly connects the PRR CERK1 with the MAPK kinase kinase MAPKKK5, establishing a link between PRRs and activation of intracellular MAPK cascades (Yamada et al., 2016). However, the fact that the influx of Ca^2+^ across the plasma membrane is nevertheless required for the PAMP-induced ROS burst and MAPK activation (Segonzac et al., 2011; Kadota et al., 2014), suggests that calcium-based regulation is also required for the ultimate activation of RBOHD and MAPKKK5. Successful initiation of signaling leads to characteristic transcriptional reprogramming. Microbial perception alters the expression of approximately 10% of the whole plant transcriptome (Navarro et al., 2004; Moore et al., 2011). This results in the production of antimicrobial compounds and cell wall-reinforcing materials around the infection site (Hahlbrock et al., 2003), which is supposed to be critical for plant immunity.

Type III effectors contribute collectively to bacterial pathogenesis by targeting host defense pathways (Katagiri et al., 2002). Effectors hijack multiple cellular processes including phytohormone signaling, proteasome-dependent protein degradation, cytoskeleton formation, manipulation of stomatal openings, establishment of intercellular apoplastic living spaces, and vesicle transport (Buttner, 2016; Toruno et al., 2016). Among all these functions, suppression of PTI has emerged as a primary role of bacterial effectors to ensure pathogenesis. Multiple effectors directly target PRRs, their co-receptors, RLCKs and MAPKs in a redundant manner and by multiple strategies to ensure suppression (Macho and Zipfel, 2015; Buttner, 2016). Some examples include the effectors AvrPto, AvrPtoB, HopAO1, and HopF2, which act as direct suppressors of PRRs such as FLS2 and/or their co-receptors BAK1 and CERK1; HopAR1 that targets BIK1 and other RLCKs; and HopAI1 and HopF2 that suppress MAPK signaling by directly modifying these proteins (Macho and Zipfel, 2015; Buttner, 2016; Toruno et al., 2016). Despite the success in identifying effectors using functional and genetic analysis, the modes of action of most of them are yet unknown.

The Gram-negative bacterial species *Pseudomonas syringae* (*P. syringae*) comprises at least 50 pathovars that can be distinguished by their host ranges (Sawada et al., 1999). For example, *P. syringae* pv. *tomato* (*Pto*) DC3000 infects tomato and can cause disease on *Arabidopsis* whereas *P. syringae* pv. *tabaci* (*Pta*) 11528 is infectious on *Nicotiana tabacum* and can grow to high levels on the model species *Nicotiana benthamiana* (*N. benthamiana*). The *Arabidopsis-Pto* DC3000 system represents the primary model for plant–bacteria interactions (Katagiri et al., 2002). In contrast, the *Pta* 11528-*N. benthamiana* system represents an interaction that offers complementary benefits to *Arabidopsis*, including high amenability to *Agrobacterium tumefaciens*-mediated transient expression of foreign genes and virus-induced gene silencing (VIGS) for gene knockdowns (Goodin et al., 2008). *Pto* DC3000 encodes nearly 30 effectors whereas *Pta* 11528 carries approximately 20 genes with homology to previously described effectors (Chang et al., 2005; Vinatzer et al., 2005; Ferreira et al., 2006; Lindeberg et al., 2006; Schechter et al., 2006; Studholme et al., 2009). Several of these *Pto* DC3000 effectors are conserved in *Pta* 11528 but it is currently unknown whether they function similarly to their *Pto* DC3000 homologs. We currently lack an integrated view of which defense pathways are suppressed by the effector repertoire, how many effectors act redundantly, and the conservation of effector function among homologs in different *Pseudomonas* strains. Also, although most of the current work has focused on defense responses elicited by flagellin, *Pseudomonas* contains many more PAMPs which may stimulate alternate pathways. It is not clear whether different PRRs feed into a limited number of signal transduction pathways, or if effectors target certain pathways specifically or act more broadly. Thus, we require a more integrative view of global molecular activities and the range of suppression by bacterial effectors to understand how host processes normally prevent successful infection.

In this study, we transiently expressed type III effector genes in leaves of *N. benthamiana* to screen and identify those with the ability to suppress defense responses. We analyzed nearly all *Pto* DC3000 effectors and most *Pta* 11528 secreted proteins for the ability to interfere with effector induced cell death, a typical hallmark of effector recognition by plant *R* genes. Secondly, we analyzed their capacities to abolish a broad range of PAMP responses including changes in cytoplasmic Ca^2+^ levels, production of ROS and induction of defense genes. Strikingly, among the 32 effectors tested, 29 showed suppressive activity in at least one of our assays. This observation is consistent with the hypothesis that one of the most important roles of effector proteins is to suppress host immunity. This work helps to identify *P. syringae* effectors that are capable of suppressing plant defenses, and to define the spectrum of their activities.

## Materials and Methods

### Elicitors

Crab shell chitin and flg22 peptide (CKANSFREDRNEDREV) were purchased from Sigma (United Kingdom) and Peptron (South Korea), respectively. *Pto* DC3000, *Pta* 11528, and *A. tumefaciens* (strain C58C1) were grown in L-medium at 28°C on a rotary shaker. Crude *A. tumefaciens*, *Pto* DC3000 and *Pta* 11528 bacterial suspensions were prepared by centrifuging overnight cultures and resuspending bacterial pellets in water.

### Statistical Methods

Statistical significance based on *t*-test analysis was developed by the GraphPad Prism program. Eight independent samples were used to analyze the significance of ROS and Ca^2+^ generation assays. For quantitative gene expression analysis, statistical significance was calculated from three independent samples.

### Bacterial Strains

*Agrobacterium tumefaciens* strain C58C1 was used for transient assays transformed with pGWB14 (*Pto* DC3000 and *Pta* 11528 effector libraries) (Nakagawa et al., 2007). Both the *Pto* DC3000 and *Pta* 11528 effector libraries were described previously (Chang et al., 2005; Gimenez-Ibanez et al., 2014). The *Pta* 11528 effector library contains individual effector genes under control of the *35S* promoter fused in-frame to a sequence encoding three C-terminal hemagglutinin (HA) epitope tags (Gimenez-Ibanez et al., 2014). In contrast, the full-length *Pto* DC3000 effectors used in this study were recombined into pDONR207 (Invitrogen) (Chang et al., 2005). These plasmids, a Gateway BP II kit (Invitrogen), and the pGWB14 (Nakagawa et al., 2007) destination vector were used to generate expression constructs in which each *Pto* DC3000 effector gene is under the control of the *35S* promoter and fused to a sequence encoding three HA epitope tags. These constructs were verified by DNA sequencing and then transferred to *A. tumefaciens* strain C58C1. Additional bacterial strains used in this study were *Pseudomonas syringae* pv. *tomato* (*Pto*) DC3000, *Pto* DC3000 *hrcC*, *Pto* DC3000Δ*avrPto, Pto* DC3000Δ*avrPtoB*, and *Pto* DC3000Δ*avrPto*Δ*avrPtoB* (Lin and Martin, 2005).

### *A. tumefaciens*-Mediated Transient Expression Assays

For transient gene expression, *A. tumefaciens* C58C1 was syringe infiltrated in *N. benthamiana* leaves at OD_600_ = 0.3–0.5 in 10 mM MgCl_2_ and 10 mM MES. Samples were collected 2 days post-inoculation for ROS, Ca^2+^, MAPK and gene expression assays. For co-infiltration assays, each of the two *A. tumefaciens* strains containing the respective effector gene were prepared at OD_600_ = 0.5 in 10 mM MgCl_2_ and 10 mM MES and then mixed in equal volumes. This solution was infiltrated in *N. benthamiana* leaves and phenotypes were scored between 1 to 7 days post-inoculation as indicated in the text.

### Virus-Induced Gene Silencing (VIGS)

Virus-induced gene silencing was performed using a tobacco rattle virus vector as previously described (Peart et al., 2002).

### Measurement of Reactive Oxygen Species (ROS) Generation

Leaf disks (0.38 cm^2^) were floated on water overnight and ROS released by the leaf tissue were measured using a chemiluminescent assay (Keppler et al., 1989). The water was replaced with 200 μl of a solution containing 20 μM luminol (Sigma, St. Louis, MO, United States) and 1 μg of horseradish peroxidase (Fluka, Buchs, Switzerland). ROS was elicited with 100 nM flg22 or 100 μg/ml of chitin in all experiments. Elicitation in the absence of any PAMP (water treatment) was included as a negative control. Luminescence was measured over a time period of 30 min using the Photek camera system (East Sussex, United Kingdom), and data were recorded as total counts.

### Measurement of Ca^2+^ Burst Generation

Transgenic *N. benthamiana* plants expressing the Ca^2+^ sensor 35S:*Aequorin* (*N. benthamiana* SLJR15) were used to measure intracellular Ca^2+^ concentrations (Segonzac et al., 2011). Leaf disks (0.38 cm^2^) were floated overnight on an aqueous 2.5 μM coelenterazine solution in the dark at room temperature. The Ca^2+^ influx was elicited with water, flg22 (100 nM) or chitin (100 μg/ml), and luminescence was measured using the Photek camera system as total counts over a time period of 30 min.

### Quantitative RT-PCR

*Nicotiana benthamiana* leaf disks expressing each individual effector were collected 2 days post-infiltration (dpi), and then floated overnight in water. Leaf disks were subsequently elicited with water (Mock EV control), 100 nM flg22 or 100 μg/ml chitin for 60 min and frozen in liquid nitrogen. Total RNA was extracted by using TRIzol-Reagent (Sigma), and the absence of genomic DNA was checked by PCR amplification of the housekeeping *NbEF1*α gene by using 2.5 μg of RNA. For analysis of gene expression, first-strand cDNA was synthesized from 2.5 μg of RNA using SuperScript RNA H-Reverse Transcriptase (Invitrogen, United Kingdom) and an oligo (dT) primer, according to the manufacturer’s instructions. For quantitative PCR, 2 μl of cDNA was combined with SYBR master mix. PCRs were performed in triplicate with a PTC-200 Peltier Thermal Cycler (MJ Research, Waltham, MA, United States), and the data were collected and analyzed with Chromo 4 Continuous Fluorescence detection system. The *NbEF1*α RNA was analyzed as an internal control and used to normalize the values for transcript abundance. All samples were related to the Mock *EV* negative control. Primers for genes used here are as follows: *NbCyp71D20*, 5′-AAGGTCCACCGCACCATGTCCTTAGAG-3′ and 5′-AAGAATTCCTTGCCCCTTGAGTACTTGC-3′; *NbACRE132*, 5′-AAGGTCCAGCGAAGTCTCTGAGGGTGA-3′ and 5′-AAGAATTC-CAATCCTAGCTCTGGCTCCTG-3′; and *NbEF1*α gene 5′-AAGGTCCAGTATGCCTGGGTGCTTGAC-3′ and 5′-AAGAATTCACAGGGACAGTTCCAATACCA-3′.

### Cell Death Assays on *N. benthamiana*

For elicitation of the HR by different *P. syringae* pathovars, overnight bacterial cultures were pelleted, resuspended in sterile 10 mM MgCl_2_ and infiltrated into the leaves of transgenic *N. benthamiana* plants at high densities (5 × 10^7^ cfu/ml). The development of cell death was scored between 1 to 4 dpi.

## Results

### Construction of Effector Libraries for *Pto* DC3000 and *Pta* 11528

*Pto* DC3000 and *Pta* 11528 encode nearly 30 and 20 effectors, respectively (Chang et al., 2005; Vinatzer et al., 2005; Ferreira et al., 2006; Lindeberg et al., 2006; Schechter et al., 2006; Studholme et al., 2009). To investigate the functions of most of these, we analyzed two libraries containing 22 *Pto* DC3000 and 10 *Pta* 11528 effector genes, respectively. Both libraries were described previously (Chang et al., 2005; Gimenez-Ibanez et al., 2014) and express each effector from a T-DNA under control of the strong 35S promoter, fused in frame to a sequence encoding three HA epitope tags. There were seven pairs of homologous effectors shared between the *Pto* DC3000 and *Pta* 11528 libraries sampled here, with varying levels of amino acid conservation. Based on protein sequence identity to their *Pto* DC3000 homologs, some *Pta* 11528 effectors were almost identical, namely HopO1-1*_Pta_*_11528_ (99%, Supplementary Figure S1) and HopT1-1*_Pta_*_11528_ (99%, Supplementary Figure S2); others showed high levels of identity such as AvrPtoB*_Pta_*_11528_ (70%, Supplementary Figure S3) and HopX1*_Pta_*_11528_ (72%, Supplementary Figure S4); whereas another group including AvrPto*_Pta_*_11528_ (42%, Supplementary Figure S5), HopI1*_Pta_*_11528_ (55%, Supplementary Figure S6) and HopF1*_Pta_*_11528_ (49%, Supplementary Figure S7) showed significantly less homology with their respective *Pto* DC3000 homologs. A complete list of the effector genes used in this study is in **Table 1**.

**Table 1:** List of *Pto* DC3000 and *Pta* 11528 effectors included in this study.

*Pto* DC3000		*Pta* 11528	
	
Effector	WB	Effector	WB
AvrPto	A	AvrPto	+
AvrPtoB	+	AvrPtoB	A
HopO1-1	+	HopT1-1	+
HopM1	+	AvrE1	A
HopN1	+	HopI1	+
HopAD1	A	HopO1-1	+
HopY1	+	HopF1	+
HopT1-1	A	HopAR1	+
HopX1	+	HopX1	A
HopC1	+	HopW1-1	+
HopF2	+		
HopAF1	+		
HopA1	+		
HopB1	+		
HopI1	+		
HopH1	+		
HopG1	+		
HopAA1-1	+		
HopK1	+		
HopQ1-1	+		
HopV1	+		
HopD1	A		


*List of *Pto* DC3000 and *Pta* 11528 effector proteins used in this study. + indicates positive detection of protein accumulation by western blotting (WB) when transiently delivered by *A. tumefaciens* in *N. benthamiana* leaves; A indicates detected effector activity in at least one assay in this study although no protein accumulation was detected.*

### Several *Pto* DC3000 and *Pta* 11528 Effectors Elicit Cell Death in *N. benthamiana*

Mutations affecting individual effector genes typically have no or only subtle effects on bacterial pathogenicity, due to redundancy among effectors (Kvitko et al., 2009). To overcome this, we used *Agrobacterium tumefaciens* to transiently express individual effector genes in *N. benthamiana* (Hann and Rathjen, 2007). Western blot analysis showed that most effector proteins accumulated to detectable levels in *N. benthamiana* (**Table 1** and Supplementary Figure S8). We detected 25 out of 32 proteins at 2 dpi. An additional seven effectors that could not be detected by western blots suppressed defense responses in at least one assay performed in this work, indicating that these proteins were expressed at undetectable levels in the plant cell. Overall, there is evidence that all *Pto* DC3000 and *Pta* 11528 effector proteins accumulated within the plant cell after transient expression.

**FIGURE 1:**
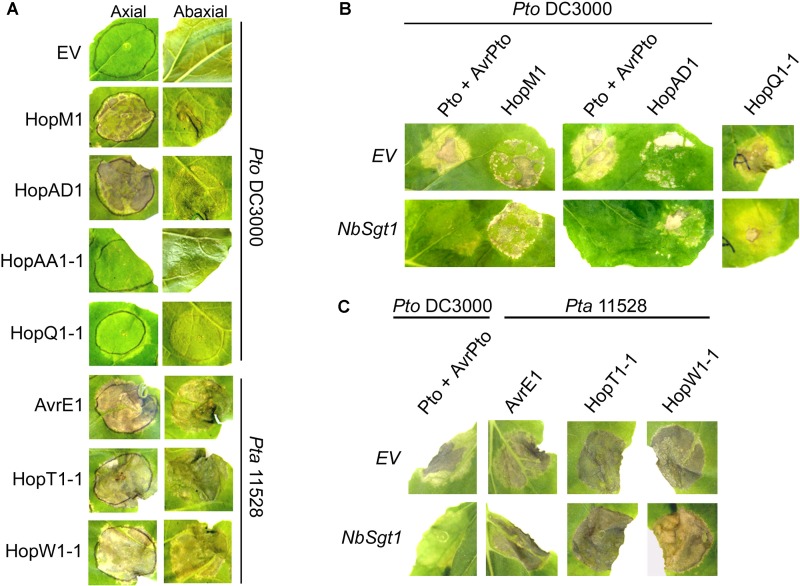
Several *Pto* DC3000 and *Pta* 11528 type-III effectors induce cell death in *N. benthamiana* leaves in an *NbSgt1*-independent manner. **(A)** Ability of *Pto* DC3000 and *Pta* 11528 effectors to elicit cell death in *N. benthamiana* when delivered by *A. tumefaciens.* Four-week-old *N. benthamiana* leaves were syringe-infiltrated with an *A. tumefaciens* C58C1 strain expressing an *EV* or each individual effector gene driven by the 35S promoter. Pictures were taken 4 days after inoculation from both sides of the plant leaf (axial and abaxial). All effector genes were tested but those that didn’t show a response are not represented. Similar results were obtained in two independent experiments. **(B)** Silencing of *NbSgt1* does not compromise cell death induced by *hopM1_Pto_*_DC3000_ and *hopAD1_Pto_*_DC3000_. Four-week-old *N. benthamiana* leaves silenced for *EV* or *NbSgt1* with a tobacco rattle virus (TRV) vector were syringe-infiltrated with *A. tumefaciens* C58C1 strains individually expressing *hopM1_Pto_*_DC3000_ or *hopAD1_Pto_*_DC3000_ effectors. *hopQ1-1_Pto_*_DC3000_, or *avrPto_Pto_*_DC3000_ co-expressed with *Pto*, were included on the same leaf as positive controls for *NbSgt1* silencing. Pictures were taken 4 days after infiltration for *hopM1_Pto_*_DC3000_, *hopAD1_Pto_*_DC3000_, and *Pto/avrPto_Pto_*_DC3000_, and 8 days after infiltration for *hopQ1-1_Pto_*_DC3000_. Similar results were obtained in three independent experiments. **(C)** Silencing of *NbSgt1* does not compromise cell death induced by *avrE1_Pta_*_11528_, *hopT1-1_Pta_*_11528_, and *hopW1-1_Pta_*_11528_ when transiently expressed in *N. benthamiana*. Four-week-old *N. benthamiana* leaves silenced for *EV* and *NbSgt1* with a TRV vector were syringe-infiltrated with *A. tumefaciens* C58C1 strains expressing one of *avrE1_Pta11528_, hopT1-1_Pta11528_*_,_ or *hopW1-1_Pta11528_* effector genes. Co-infiltrated *avrPto_Pto_*_DC3000_ and *Pto* were included on the same leaf as a positive control for *NbSgt1* silencing. Pictures were taken 4 days after inoculation. Similar results were obtained in three independent experiments.

Four *Pto* DC3000 and three *Pta* 11528 effectors elicited various forms of necrosis when transiently expressed in *N. benthamiana* leaves, whereas no phenotype was observed in control tissue expressing an *EV* construct (**Figure 1A**). Leaves when transiently expressing *hopAD1_Pto_*_DC3000_, *avrE1_Pta_*_11528_, *hopW1-1_Pta_*_11528_, or *hopT1-1_Pta_*_11528_ displayed strong cell death within two or three dpi. However, expression of *avrE1_Pta_*_11528_ induced a stronger and faster necrosis compared to expression of *hopAD1_Pto_*_DC3000_, *hopW1-1_Pta_*_11528_, and *hopT1-1_Pta_*_11528_. In contrast, expression of *hopM1_Pto_*_DC3000_ induced only a patchy necrosis within 3 to 4 dpi, whereas *hopAA1-1_Pto_*_DC3000_ led to appearance of shiny areas, obvious only on the abaxial leaf surface that never developed into full necrosis. Leaves expressing *hopQ1-1_Pto_*_DC3000_ displayed only mild chlorosis within 4 days. In our experiments, such chlorotic areas did not normally progress further into necrosis. Finally, we observed mild chlorotic areas associated with *avrPtoB_Pta_*_11528_ and *hopAR1_Pta_*_11528_ expression that appeared consistently at 7 dpi (data not shown). Effector protein accumulation did not correlate with the intensity of necrosis. For example, neither AvrE1*_Pta_*_11528_ nor HopAD1*_Pto_*_DC3000_ proteins could be detected by western blots, but they both induced fast and strong necrosis. Overall, 7 of 32 effector genes caused cell death when expressed transiently in *N. benthamiana* leaves.

Plant cell death is associated with effector recognition and subsequent HR in resistant plants as well as with the formation of lesions in susceptible plants (Alfano and Collmer, 2004). To investigate the nature of the cell death phenotype caused by individual expression of several *Pto* DC3000 and *Pta* 11528 effectors, we used VIGS to knock down the expression of *NbSgt1* in *N. benthamiana. NbSgt1* is required for NB-LRR proteins to trigger the defense-associated HR in response to effector recognition (Peart et al., 2002). Three weeks post-silencing, we transiently expressed *hopM1_Pto_*_DC3000_, *hopAD1_Pto_*_DC3000_, *hopQ1-1_Pto_*_DC3000,_ *avrE1_Pta_*_11528_, *hopT1-1_Pta_*_11528_, and *hopW1-1_Pta_*_11528_ in *EV*- or *NbSgt1*-silenced plants. Because of the difficulty in scoring the mild phenotype of *hopAA1-1_Pto_*_DC3000_ expression, we excluded this gene from the assay. In addition, we co-expressed *avrPto_Pto_*_DC3000_ and the protein kinase gene *Pto* as a control. Pto in complex with the NB-LRR protein Prf recognizes AvrPto*_Pto_*_DC3000_ *in vivo*, leading to induction of a *NbSgt1*-dependent HR (Scofield et al., 1996; Tang et al., 1996; Peart et al., 2002; Mucyn et al., 2006). As expected, silencing of *NbSgt1* compromised the cell death phenotype developed by the AvrPto*_Pto_*_DC3000_/Pto control in *N. benthamiana*, and also by *hopQ1-1_Pto_*_DC3000,_ which was reported previously to be *NbSgt1*-dependent (Wei et al., 2007). In contrast, cell death induced by any of the *Pto* DC3000 or *Pta* 11528 effectors was unaffected in both *EV-* and *NbSgt1-*silenced plants (**Figures 1B,C**). Thus, the necrosis induced by *hopM1_Pto_*_DC3000_, *hopAD1_Pto_*_DC3000_, *avrE1_Pta_*_11528_, *hopT1-1_Pta_*_11528_, and *hopW1-1_Pta_*_11528_ was not due to recognition by plant *R* genes. Taken together, these results suggest that *NbSgt1* does not play a role in the cell death induced by these effectors.

### *Pto* DC3000 and *Pta* 11528 Effectors Redundantly Suppress *NbSgt1*-Independent Effector-Induced Cell Death

**FIGURE 2:**
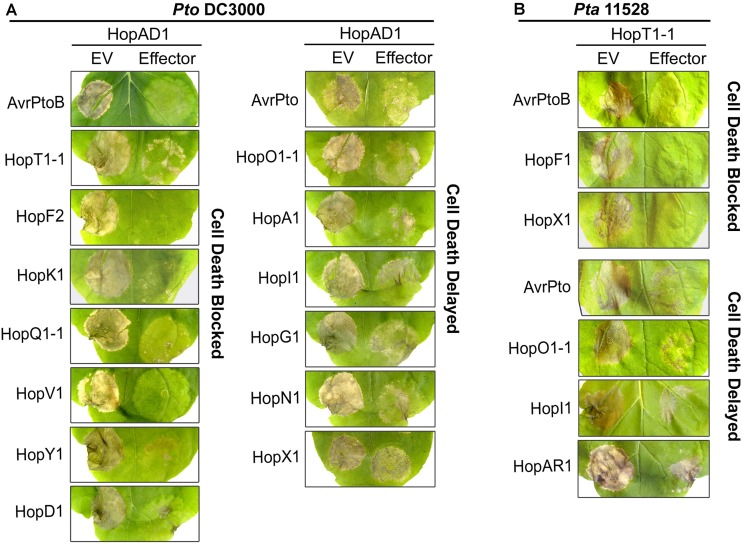
Most *Pto* DC3000 and *Pta* 11528 effectors can interfere with effector-induced cell death. **(A)** Suppression of *hopAD1_Pto_*_DC3000_ induced cell death by *Pto* DC3000 effectors. The effector *hopAD1_Pto_*_DC3000_ was co-expressed in *N. benthamiana* leaves with each effector in the *Pto* DC3000 repertoire, or an *EV* control. Each of the two *A. tumefaciens* strains containing the respective effector gene or an EV control were prepared at OD_600_ = 0.5 then mixed in equal volumes. Cell death induced by *hopAD1_Pto_*_DC3000_ was scored 4 days after infiltration. Similar results were obtained in three independent experiments. **(B)** Suppression of *hopT1-1_Pta_*_11528_ induced cell death by the *Pta* 11528 effector repertoire. *hopT1-1_Pta_*_11528_ was co-expressed with each individual effector in the *Pta* 11528 repertoire or an *EV* control in *N. benthamiana*. Pictures were taken 4 days after inoculation and similar results were obtained in three independent experiments.

Some effector proteins can mask the ability of other effectors to trigger cell death (Jackson et al., 1999; Jamir et al., 2004; Guo et al., 2009). We investigated whether the cell death phenotypes induced by certain *Pto* DC3000 or *Pta* 11528 effectors could be suppressed by others encoded by these pathovars. We transiently co-delivered the necrosis-inducing effector *hopAD1_Pto_*_DC3000_ with each of the remaining effectors of the *Pto* DC3000 library. Among the 21 genes screened, 8 effectors completely blocked *hopAD1_Pto_*_DC3000_ induced cell death, whereas 7 additional genes significantly delayed the timing of necrosis appearance (**Figure 2A**). Effector genes that completely blocked cell death included *avrPtoB_Pto_*_DC3000_, *hopT1-1_Pto_*_DC3000_, *hopF2_Pto_*_DC3000_, *hopK1_Pto_*_DC3000_, *hopQ1-1_Pto_*_DC3000_, *hopV1_Pto_*_DC3000_, *hopY1_Pto_*_DC3000_, and *hopD1_Pto_*_DC3000_. In addition, effector genes that delayed the appearance of cell death were *avrPto_Pto_*_DC3000_, *hopO1-1_Pto_*_DC3000_, *hopA1_Pto_*_DC3000_, *hopI1_Pto_*_DC3000_, *hopG1_Pto_*_DC3000_, *hopN1_Pto_*_DC3000_, and *hopX1_Pto_*_DC3000_. These effectors interfered not only with *hopAD1_Pto_*_DC3000_ but also with *hopM1_Pto_*_DC3000_ induced necrosis, with the exception of *hopX1_Pto_*_DC3000_ (Supplementary Figure S9). To test whether a similar activity is present in the *Pta* 11528 effector inventory, we screened these genes for the capacity to suppress *hopT1-1_Pta_*_11528_ induced cell death. Similar to previous results, most *Pta* 11528 effectors interfered with necrosis induced by this protein (**Figure 2B**). Expression of *avrPtoB_Pta_*_11528_, *hopF1_Pta_*_11528_, *hopX1_Pta_*_11528_ completely suppressed *hopT1-1_Pta_*_11528_ induced cell death, whereas *avrPto_Pta_*_11528_, *hopO1-1_Pta_*_11528_, *hopI1_Pta_*_11528_, and *hopAR1_Pta_*_11528_ delayed it. We next investigated whether the cell death phenotypes induced by the *Pto* DC3000 effectors *hopAD1* and *hopM1* could be suppressed by an effector encoded within the *Pta* 11528 repertoire, such as *avrPtoB*. The effector *avrPtoB_Pta_*_11528_ also interfered with the cell death phenotypes induced by both *hopAD1_Pto_*_DC3000_ and *hopM1_Pto_*_DC3000_ (Supplementary Figure S10). Therefore, our data indicate that the vast majority of *Pto* DC3000 and *Pta* 11528 effectors can interfere with cell death induced by certain effectors within the same bacterial pathovar and in the case of *avrPtoB_Pta_*_11528_, also across bacterial pathovars.

We next analyzed the ability of *Pto* DC3000 effectors to antagonize specific *R* gene-dependent HR events in *N. benthamiana*. In tomato, the unrelated effectors AvrPto*_PtoDC3000_* and AvrPtoB*_PtoDC3000_* trigger disease resistance in plants carrying Pto and Prf (Scofield et al., 1996; Tang et al., 1996; Kim et al., 2002; Mucyn et al., 2006). Thus, we next assessed the ability of transgenic *N. benthamiana* plants expressing the tomato *Pto* and *Prf* genes (*N. benthamiana* R411A) (Balmuth and Rathjen, 2007) for the ability to trigger HR upon infiltration of high bacterial densities of *Pto* DC3000, or isogenic strains lacking *avrPto*_*PtoDC*3000_ (*Pto* DC3000Δ*avrPto*), *avrPtoB*_*PtoDC*3000_ (*Pto* DC3000Δ*avrPtoB*) or both (*Pto* DC3000Δ*avrPto*Δ*avrPtoB*). We additionally included a *Pto* DC3000 strain lacking a functional TTSS required for effector secretion as a negative control (*Pto* DC3000 *hrcC*). Three days post-inoculation, *Pto* DC3000, *Pto* DC3000Δ*avrPto*, and *Pto* DC3000Δ*avrPtoB* elicited strong HRs (Supplementary Figure S11), indicating that the effectors secreted by *Pto* DC3000 do not interfere with the AvrPto*_PtoDC3000_* and/or AvrPtoB*_PtoDC3000_*-dependent recognition by Pto/Prf.

### Multiple *Pto* DC3000 and *Pta* 11528 Effectors Target PAMP(s) Signaling Pathways at Different Steps

To identify suppressive effectors, we screened our collection for the ability to suppress the PAMP-dependent Ca^2+^ and ROS bursts, and activation of defensive marker genes in *N. benthamiana*. For these experiments, we used two PAMPs; the flg22 peptide derived from bacterial flagellin (Chinchilla et al., 2007), and the oligosaccharide chitin which is a component of fungal cell walls. These two PAMPs are structurally unrelated and perceived by different receptor complexes (Boutrot and Zipfel, 2017). Each effector was expressed individually in *N. benthamiana* for 2 days and the harvested tissue subjected to treatments with either flg22 or chitin followed by one of the three assays. The first was a ROS assay done in wild-type (WT) *N. benthamiana* leaves. The second assayed for an increase in cytosolic Ca^2+^ concentrations using transgenic *N. benthamiana* plants expressing the 35S:*Aequorin* reporter (*N. benthamiana* SLJR15) (Segonzac et al., 2011). Finally, we assayed induction of the well-characterized defense genes *NbCyp71D20* and *NbACRE132* by quantitative RT-PCR (Navarro et al., 2004; Segonzac et al., 2011). The majority of *Pto* DC3000 and *Pta* 11528 effectors interfered with PAMP-induced responses and could be divided into three major groups based on their range of suppressive activities. Firstly, broad-range suppressors of all tested PAMP-induced early responses (Group A). Secondly, suppressors of the PAMP-induced ROS burst, without affecting Ca^2+^ influx (Group B). And finally, effectors that suppressed PAMP-induced transcriptional activation of defense genes without affecting other outputs (Group C).

#### Group A: Broad-Range Suppressors of PAMP-Induced Early Responses

**FIGURE 3:**
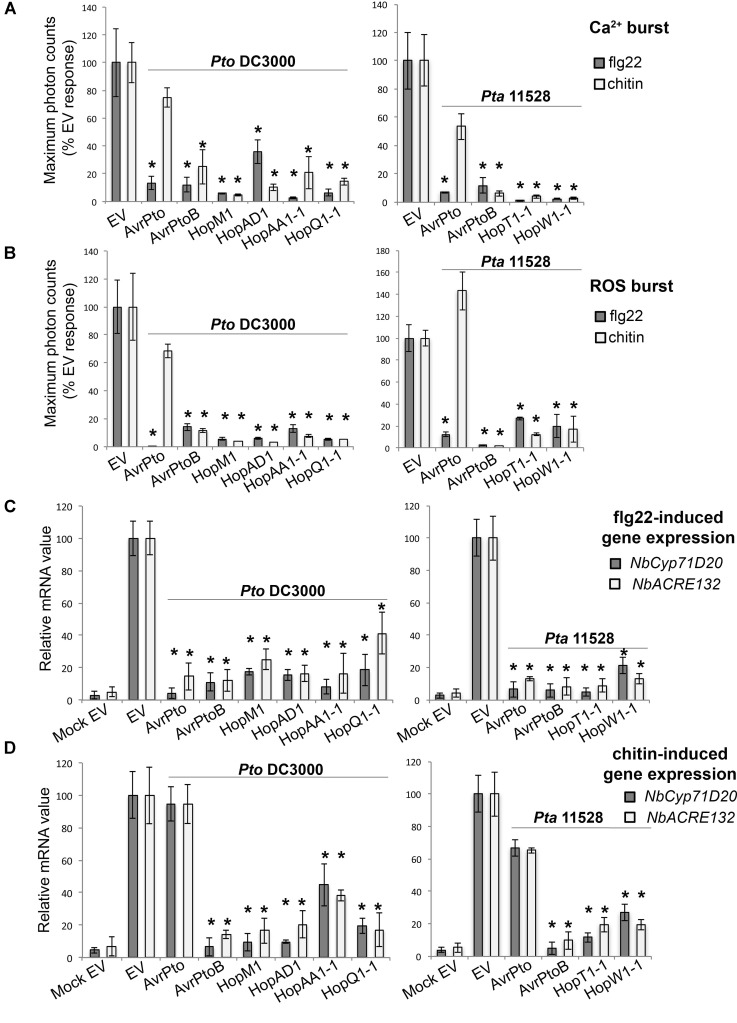
Group A effectors: broad-range suppressors of early PAMP-induced responses. **(A)** Ca^2+^ burst in *N. benthamiana* SLJR15 transgenic plants transiently expressing an *EV* or each Group A effector as indicated after treatment with 100 nM flg22 or 100 μg/ml chitin. Data are presented relative (%) to control EV (PAMP-treated). Error bars represent the standard error of the mean (SEM; *n* = 8). Statistical significance compared to *N. benthamiana* tissue transiently expressing *EV* is indicated by asterisks (Student’s *t*-test, ^∗^*p* ≤ 0.01). **(B)** ROS burst in *N. benthamiana* plants transiently expressing an *EV* or each Group A effector from *Pto* DC3000 or *Pta* 11528 after treatment with 100 nM flg22 or 100 μg/ml chitin. Data are presented relative (%) to control EV (PAMP-treated). Error bars represent the standard error of the mean (SEM; *n* = 8). Statistical significance compared to *N. benthamiana* tissue transiently expressing *EV* is indicated by asterisks (Student’s *t*-test, ^∗^*p* ≤ 0.01). **(C)** Quantitative RT-PCR analysis of *NbCyp71D20* and *NbACRE132* gene expression 60 min after treatment with 100 nM flg22 in *N. benthamiana* leaf tissue transiently expressing an *EV* control or each Group A effector protein as indicated. A mock induction treatment in *N. benthamiana* leaf tissue transiently expressing *EV* (Mock EV) is included as a negative control. All samples were normalized against the housekeeping gene *NbEF1*α and the measurements represent the ratio of expression levels (%) compared to the flg22-induced EV sample. Error bars represent (SEM; *n* = 3). Statistical significance compared to PAMP-induced *N. benthamiana* tissue expressing *EV* is indicated by asterisks (Student’s *t*-test, ^∗^*p* ≤ 0.01). **(D)** Quantitative RT-PCR analysis of *NbCyp71D20* and *NbACRE132* defense gene expression 60 min after treatment with 100 μg/ml chitin in *N. benthamiana* leave tissue transiently expressing an *EV* control or each Group A effector protein as indicated. A mock induction treatment in *N. benthamiana* leaf tissue transiently expressing *EV* (Mock EV) is included as a negative control. All samples were normalized against the housekeeping gene *NbEF1*α and the measurements represent the ratio of expression levels (%) compared to the chitin-induced EV sample. Error bars represent (SEM; *n* = 3). Statistical significance compared to PAMP-induced *N. benthamiana* tissue expressing *EV* is indicated by asterisks (Student’s *t*-test, ^∗^*p* ≤ 0.01). The results shown in **(A–D)** are representative of three independent experiments. All effector genes were tested but those that didn’t show any response of suppression are not represented.

As expected, leaf tissue expressing an *EV* control and treated with either flg22 or chitin led to an increase in cytosolic Ca^2+^ levels (**Figure 3A**), the production of ROS (**Figure 3B**) and the strong induction of both *NbCyp71D20* and *NbACRE132* defensive marker genes (**Figures 3C,D**). We found that six *Pto* DC3000 and four *Pta* 11528 effectors compromised the activation of all tested responses to both PAMPs simultaneously (**Figure 3**). These effectors included the *Pto* DC3000 effectors AvrPto*_Pto_*_DC3000_, AvrPtoB*_Pto_*_DC3000_, HopM1*_Pto_*_DC3000_, HopAD1*_Pto_*_DC3000_, HopAA1-1*_Pto_*_DC3000_ and HopQ1-1*_Pto_*_DC3000_, and the *Pta* 11528 effectors AvrPto*_Pta_*_11528_, AvrPtoB*_Pta_*_11528_, HopT1-1*_Pta_*_11528_, and HopW1-1*_Pta_*_11528._ AvrE*_Pta_*_11528_ was omitted from these experiments because of the strong cell death phenotype caused by its expression. Interestingly, in contrast to the other effectors of this group, AvrPto*_Pto_*_DC3000_ and AvrPto*_Pta_*_11528_ only mildly suppressed or had no effect on chitin-induced defense responses, while they strongly compromised all flg22-induced outputs. Overall, 10 effectors from *Pto* DC3000 and *Pta* 11528 acted as early broad-range suppressors of PAMP-induced responses. These results suggest that multiple specificities and strategies may converge for simultaneous suppression of early PAMP-triggered immunity elicited by multiple microbial elicitors.

#### Group B: Suppressors of PAMP-Induced ROS Burst

**FIGURE 4:**
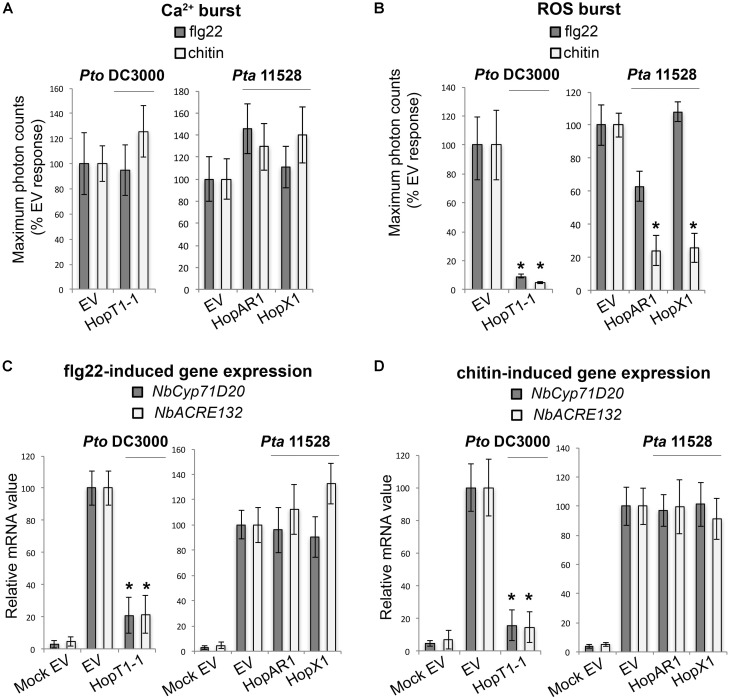
Group B effectors: suppressors of PAMP-induced ROS burst. **(A)** Ca^2+^ burst in *N. benthamiana* SLJR15 transgenic plants transiently expressing an empty *EV* or each Group B effector as indicated after treatment with 100 nM flg22 or 100 μg/ml chitin. Data are presented relative (%) to control EV (PAMP-treated). Error bars represent the standard error of the mean (SEM; *n* = 8). Statistical significance compared to *N. benthamiana* tissue transiently expressing *EV* is indicated by asterisks (Student’s *t*-test, ^∗^*p* ≤ 0.01). **(B)** ROS burst in *N. benthamiana* plants transiently expressing an *EV* or each Group B effector as indicated after treatment with 100 nM flg22 or 100 μg/ml chitin. Data are presented relative (%) to control EV (PAMP-treated). Error bars represent the standard error of the mean (SEM; *n* = 8). Statistical significance compared to *N. benthamiana* tissue transiently expressing *EV* is indicated by asterisks (Student’s *t*-test, ^∗^*p* ≤ 0.01). **(C)** Quantitative RT-PCR analysis of *NbCyp71D20* and *NbACRE132* defense gene expression 60 min after treatment with 100 nM flg22 in *N. benthamiana* leaf tissue transiently expressing an *EV* control or each Group B effector as indicated. A mock induction treatment in *N. benthamiana* leaf tissue transiently expressing *EV* (Mock EV) is included as a negative control. All samples were normalized against the housekeeping gene *NbEF1*α and the measurements represent the ratio of expression levels (%) compared to the flg22-induced EV sample. Error bars represent (SEM; *n* = 3). Statistical significance compared to PAMP-induced *N. benthamiana* tissue expressing *EV* is indicated by asterisks (Student’s *t*-test, ^∗^*p* ≤ 0.01). **(D)** Quantitative RT-PCR analysis of *NbCyp71D20* and *NbACRE132* defense gene expression 60 min after treatment with 100 μg/ml chitin in *N. benthamiana* leaf tissue transiently expressing an *EV* control or each Group A effector as indicated. A mock induction treatment in *N. benthamiana* leaf tissue transiently expressing *EV* (Mock EV) is included as a negative control. All samples were normalized against the housekeeping gene *NbEF1*α and the measurements represent the ratio of expression levels (%) compared to the chitin-induced EV sample. Error bars represent (SEM; *n* = 3). Statistical significance compared to PAMP-induced *N. benthamiana* tissue expressing *EV* is indicated by asterisks (Student’s *t*-test, ^∗^*p* ≤ 0.01). The results shown in **(A–D)** are representative of three independent experiments. All effector genes were tested but those that didn’t show any response of suppression are not represented.

Apoplastic generation of ROS upon microbial perception plays a key role in the activation of disease resistance mechanisms in plants (Yoshioka et al., 2008). We found three effectors, HopT1-1*_Pto_*_DC3000_, HopX1*_Pta_*_11528_, and HopAR1*_Pta_*_11528_ that suppressed PAMP-induced ROS production but not the Ca^2+^ influx (**Figures 4A,B**). HopT1-1*_Pto_*_DC3000_ did not distinguish between flg22 and chitin, whereas HopX1*_Pta_*_11528_ and HopAR1*_Pta_*_11528_ suppressed chitin-induced ROS more efficiently than flg22 ROS. Only HopT1-1*_Pto_*_DC3000_ suppressed induction of the defense markers *NbCyp71D20* and *NbACRE132* by both elicitors, whereas HopX1*_Pta_*_11528_ and HopAR1*_Pta_*_11528_ did not suppress defense gene induction (**Figures 4C,D**). We designated these Group B effectors, based on their ability to suppress the ROS burst upon PAMP perception without interfering with the Ca^2+^ influx. The specificity of HopX1*_Pta_*_11528_ and HopAR1*_Pta_*_11528_ for suppression of chitin-induced ROS suggests that the chitin and flg22 perception systems may differ in some key components required for ROS production. It is remarkable that HopT1-1*_Pto_*_DC3000_ and HopX1*_Pta_*_11528_ behaved differently from their respective *Pta* 11528 and *Pto* DC3000 homologs. Compared to HopT1-1*_Pto_*_DC3000_, HopT1-1*_Pta11528_* acted as a broad-range suppressor of early PAMP-induced responses (Group A), whereas HopX1*_Pto_*_DC3000_ did not compromise the chitin-triggered ROS burst that was typically suppressed in the presence of HopX1*_Pta_*_11528_. The capacity of several effectors to suppress ROS production upon PAMP sensing supports the importance of apoplastic ROS production in immunity to bacterial pathogens.

#### Group C: Suppressors of PAMP-Induced Transcriptional Activation of Defense Genes

**FIGURE 5:**
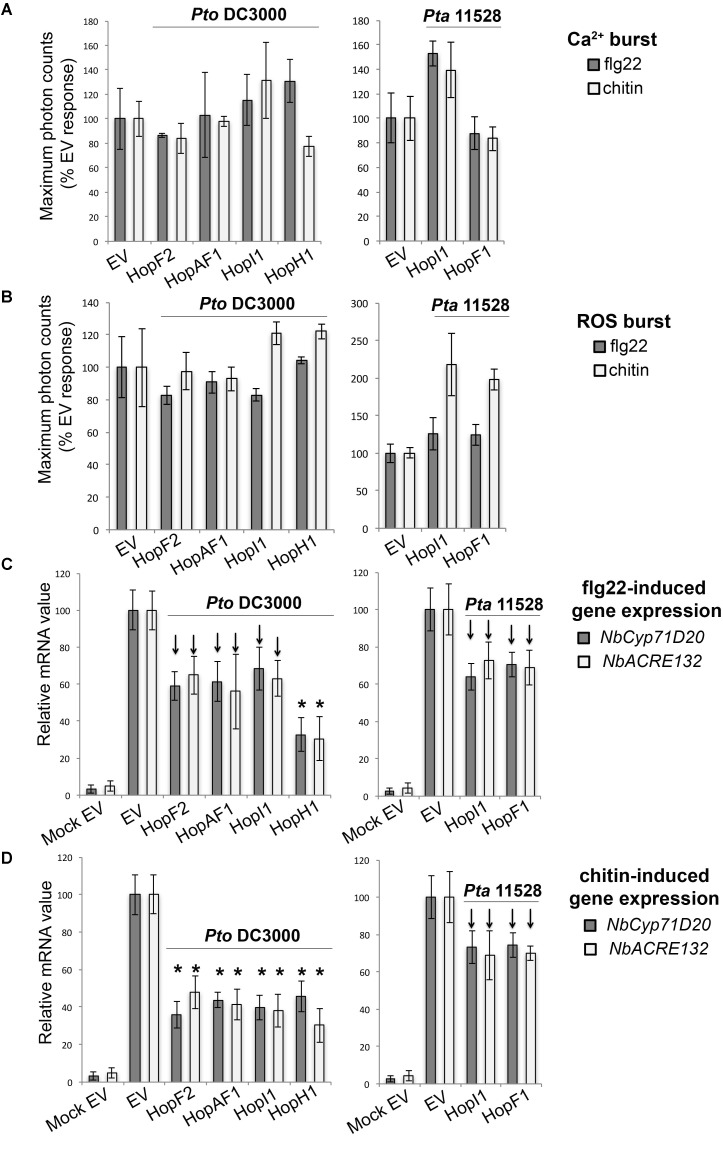
Group C effectors: suppressors of PAMP-induced transcriptional activation of defense genes. **(A)** Ca^2+^ burst in *N. benthamiana* SLJR15 transgenic plants transiently expressing an *EV* or each Group C effector as indicated after treatment with 100 nM flg22 or 100 μg/ml chitin. Data are presented relative (%) to control EV (PAMP-treated). Error bars represent the standard error of the mean (SEM; *n* = 8). Statistical significance compared to *N. benthamiana* tissue transiently expressing *EV* is indicated by asterisks (Student’s *t*-test, ^∗^*p* ≤ 0.01). **(B)** ROS burst in *N. benthamiana* plants transiently expressing an *EV* or each Group C effector as indicated after treatment with 100 nM flg22 or 100 μg/ml chitin. Data are presented relative (%) to control EV (PAMP-treated). Error bars represent the standard error of the mean (SEM; *n* = 8). Statistical significance compared to *N. benthamiana* tissue transiently expressing *EV* is indicated by asterisks (Student’s *t*-test, ^∗^*p* ≤ 0.01). **(C)** Quantitative RT-PCR analysis of *NbCyp71D20* and *NbACRE132* defense gene expression 60 min after treatment with 100 nM flg22 in **N. benthamiana* leaf tissue transiently expressing an *EV* control or each Group C effector as indicated. A mock induction treatment in *N. benthamiana* leaf tissue transiently expressing *EV* (Mock EV) is included as a negative control. All samples were normalized against the housekeeping gene *NbEF1*α and the measurements represent the ratio of expression levels (%) compared to the flg22-induced EV sample. Error bars represent (SEM; *n* = 3). Statistical significance compared to PAMP-induced *N. benthamiana* tissue expressing *EV* is indicated by asterisks (Student’s *t*-test, ^∗^*p* ≤ 0.01). Arrows indicate consistent reduction observed in independent experiments. **(D)** Quantitative RT-PCR analysis of *NbCyp71D20* and *NbACRE132* defense gene expression 60 min after treatment with 100 μg/ml chitin in *N. benthamiana* leaf tissue transiently expressing an *EV* control or each Group C effector proteins as indicated. A mock induction treatment in *N. benthamiana* leaf tissue transiently expressing *EV* (Mock EV) is included as a negative control. All samples were normalized against the housekeeping gene *NbEF1*α and the measurements represent the ratio of expression levels (%) compared to the chitin-induced EV sample. Error bars represent (SEM; *n* = 3). Statistical significance compared to PAMP-induced *N. benthamiana* tissue expressing *EV* is indicated by asterisks (Student’s *t*-test, ^∗^*p* ≤ 0.01). Arrows indicate consistent reduction observed in independent experiments. The results shown in (A–D) are representative of three independent experiments. All effector genes were tested but those that didn’t show any response of suppression are not represented.*

Recognition of microbial elicitors orchestrates an extensive defense-oriented transcriptional reprogramming of the affected cell (Navarro et al., 2004; Wan et al., 2008). In this study, we identified six effectors which downregulated flg22- and chitin-induced transcriptional activation of *NbCyp71D20* and *NbACRE132* genes. These included the *Pto* DC3000 effectors HopF2*_Pto_*_DC3000,_ HopAF1*_Pto_*_DC3000_, HopI1*_Pto_*_DC3000_, and HopH1*_Pto_*_DC3000_, and the *Pta* 11528 effectors HopF1*_Pta_*_11528_ and HopI1*_Pta_*_11528_. These effectors reduced rather than abolished gene induction, to a range of about 40–70% of the *EV* controls. Each effector caused very similar reductions in the expression of each marker gene. None of them compromised the Ca*^2+^* and ROS bursts produced after flg22 or chitin perception (**Figures 5A,B**). Among them, HopH1*_Pto_*_DC3000_ showed the strongest ability to compromised flg22- and chitin-induced expression of both *NbCyp71D20* and *NbACRE132* genes (**Figures 5C,D**), whereas the remaining effectors showed reproducibly intermediate levels of suppression. HopF1*_Pta_*_11528_ and HopI1*_Pta_*_11528_ consistently reduced flg22- and chitin-induced gene expression to a similar extent as their *Pto* DC3000 homologs. The fact that effector proteins from both *Pto* DC3000 and *Pta* 11528 compromised PAMP-induced gene expression shows that this function is conserved between strains. In all cases, these effectors compromised transcriptional responses to both flg22 and chitin, suggesting that these pathways converge at some point downstream of each PRR complex.

### HopAD1 and HopM1 Compromise PAMP-Dependent ROS Production in the Presence of the Cell Death Blocker HopY1

**FIGURE 6:**
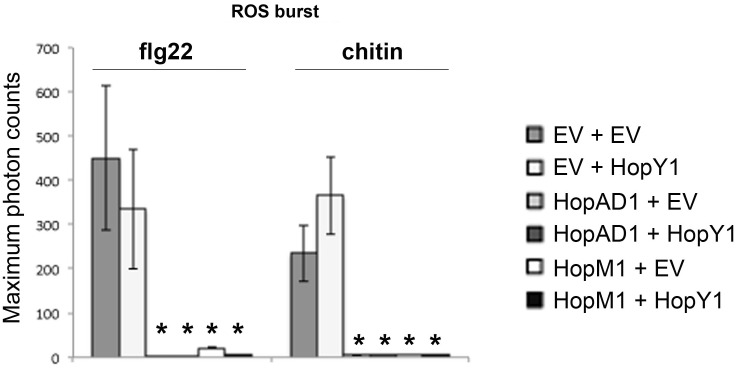
HopAD1 and HopM1 compromise PAMP-dependent ROS production in the presence of the cell death blocker HopY1. ROS burst in *N. benthamiana* plants transiently co-expressing an empty vector (*EV*), *hopAD1_Pto_*_DC3000_ or *hopM1_Pto_*_DC3000_ with the effector *hopY1_Pto_*_DC3000_ or the *EV* control as indicated after treatment with 100 nM flg22 or 100 μg/ml chitin. Error bars represent the standard error of the mean (SEM; *n* = 8). Statistical significance compared to *N. benthamiana* tissue transiently co-expressing *EV* (*EV + EV.* PAMP-treated) is indicated by asterisks (Student’s *t*-test, ^∗^*p* ≤ 0.01). The result shown is representative of two independent experiments.

Some *Pto* D3000 effectors both suppress PAMP responses and elicit cell death when expressed transiently in *N. benthamiana* leaves. In order to determine whether cell death caused the absence of PAMP-induced defense responses, we suppressed the *hopAD1_Pto_*_DC3000_ and *hopM1_Pto_*_DC3000_ induced necroses by co-expressing them with the previously described effector *hopY1_Pto_*_DC3000_ in *N. benthamiana*. This effector blocked the necroses triggered by *hopAD1_Pto_*_DC3000_ and *hopM1_Pto_*_DC3000_, but did not compromise PAMP responses in any of our assays. Both *hopAD1_Pto_*_DC3000_ and *hopM1_Pto_*_DC3000_ suppressed the ROS burst upon flg22 and chitin treatment to the same extent independent of the presence of *hopY1_Pto_*_DC3000_ (**Figure 6**), suggesting that ROS suppression upon PAMP treatment by these effectors is likely the outcome of their activities within the plant cell rather than due to the death of the infiltrated tissue.

## Discussion

**FIGURE 7:**
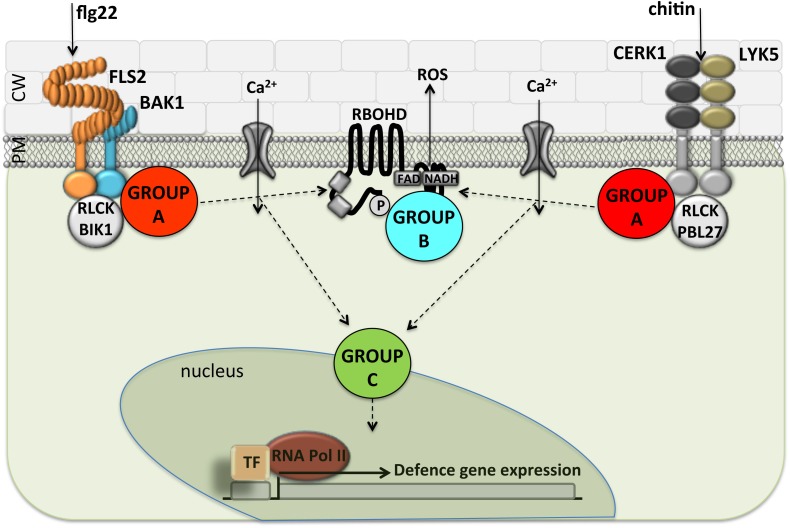
Separation of *P. syringae* type-III effectors into functional groups based on the ability to suppress different PAMP responses. The scheme represents the FLS2 and LYK5 PRR complexes at the plasma membrane and shows the major complex components. The cellular events measured here downstream of PAMP perception; Ca^2+^ influx, ROS generation and changes in defense gene activation, are depicted according to the model in Segonzac et al. (2011). As an exception, the Group A effectors AvrPto*_Pto_*_DC3000_ and AvrPto*_Pta_*_11528_ suppressed flg22-induced outputs but had no effect on chitin-induced defense responses. Arrows are indicative and drawn for conceptual simplicity to show the order of events, but junctions may exist differently from depicted. PM, plasma membrane; CW, cell wall; ROS, reactive oxygen compound synthesis; TF, transcription factor.

**FIGURE 8:**
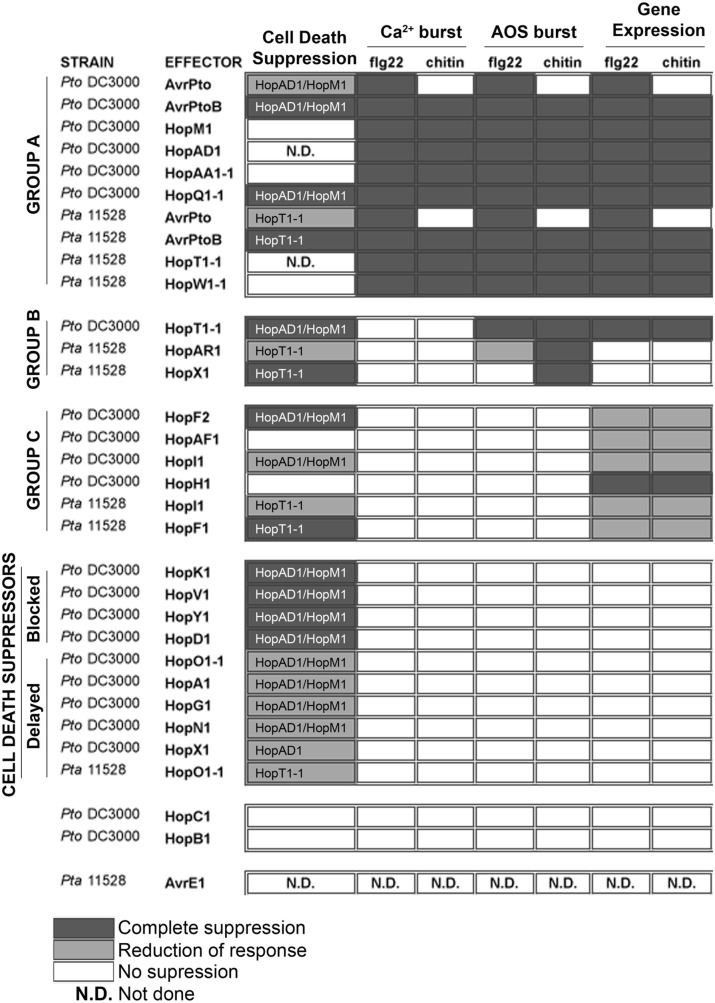
Summary of results. Summary of the ability of all *Pto* DC3000 and *Pta* 11528 effectors tested in this manuscript to interfere with effector-induced cell death in *N. benthamiana* and the activation of PAMP-dependent defense responses including the Ca^2+^ influx, generation of ROS and defense gene expression. Dark gray indicates complete suppression. Light gray indicates significant reduction compared to positive control. White indicates no suppression. ND, not done. The cell death suppression box indicates those effectors for which cell death was compromised, namely *hopAD1_Pto_*_DC3000_ or *hopM1_Pto_*_DC3000_ for *Pto* DC3000, and *hopT1-1_Pta_*_11528_ for *Pta* 11528.

Bacterial pathogens use a TTSS to deliver effector proteins into eukaryotic cells. This mechanism enables the bacterium to grow to high levels and produce disease symptoms (Collmer et al., 2002). We found that 20 of the 22 *Pto* DC3000 effectors and 9 of 10 *Pta* 11528 effectors tested in this study showed suppressive activity in at least one of defense assay when overexpressed transiently using *Agrobacterium*-mediated transformation. This is consistent with the notion that a major role of effector proteins is to suppress plant innate immunity, and that they do so at different times and points in the signaling pathways. A comprehensive picture of the activity of effector molecules inside plant cells is summarized in **Figures 7**, **8**.

### Evolutionary Aspects of Type III Effectors Conserved in Both *Pto* DC3000 and *Pta* 11528

Comparing the activities of the effector repertoires of *Pto* DC3000 and *Pta* 11528 is a useful first step toward an understanding how these bacteria cause disease on different host species. We generated a library of 10 *Pta* 11528 effector genes, which constitute about half of the effectors present in the genome (Studholme et al., 2009). Seven of these effectors have homologs in the *Pto* DC3000 effector library analyzed in this work. However, the level of conservation varies among them. Some effectors show high levels of identity, including HopO1-1*_Pta_*_11528_ and HopT1-1*_Pta_*_11528,_ which are 99 and 98% identical at the amino acid level with their respective *Pto* DC3000 homologs (Supplementary Figures S1, S2). Putative catalytic functions identified by prediction programs are present in both the *Pta* 11528 and *Pto* DC3000 effector homologs. Therefore, it seems likely that they are conserved functionally. Despite this, the transgenic expression phenotypes of the *Pta* 11528 and *Pto* DC3000 HopT1-1 homologs differed significantly. While HopT1-1*_Pta_*_11528_ elicited strong necrosis in *N. benthamiana*, HopT1-1*_Pto_*_DC3000_ did not. Although the effectors share high identity at the amino acid level, they differ in five residues that likely confer the basis for this behavior. Likewise, the cysteine protease HopX1*_Pta_*_11528_ is highly similar to its *Pto* DC3000 homolog and catalytic residues required for putative enzymatic activity are conserved (Supplementary Figure S4). However, only the *Pta* 11528 version of HopX1 interfered with the chitin-induced ROS burst. It was previously reported that HopX1*_Pta_*_11528_ but not HopX1*_Pto_*_DC3000_ targets conserved JAZ transcriptional repressors to activate jasmonate (JA) hormone signaling (Gimenez-Ibanez et al., 2014). In contrast to *Pto* DC3000, the *Pta* 11528 strain does not produce coronatine (COR), a phytotoxin that mimics the active form of JA, and therefore, exploits an alternative evolutionary strategy to activate the pathway through the HopX1*_Pta_*_11528_ effector. *HopX1* and COR biosynthetic genes generally do not co-exist in a single strain, but in the few cases where both occur, the HopX1 alleles contain mutations in functionally essential residues (Yang et al., 2017), suggesting that redundancy between COR and HopX1 might have inactivated and/or allowed the effector to evolve toward other functions. In contrast, the *Pto* DC3000 E3-ligase effector AvrPtoB also shares high level of amino acid identity (70%) with AvrPtoB*_Pta_*_11528_ (Supplementary Figure S3) and the amino acids critical for E3 ligase activity (F479A, T450, F525, and P533) are present in AvrPtoB*_Pta_*_11528_. This suggests that AvrPtoB*_Pta_*_11528_ is also an active E3-ligase in plant cells and congruently, both *Pto* DC3000 and *Pta* 11528 homologs showed similar abilities to interfere with PAMP-triggered immunity (Janjusevic et al., 2006; Ntoukakis et al., 2009). Interestingly, although AvrPto*_Pta_*_11528_, HopI1*_Pta_*_11528_, and HopF1*_Pta_*_11528_ show significantly less homology with their respective *Pto* DC3000 homologs, they showed functional conservation in PAMP-induced defense suppression assays (Supplementary Figures S5–S7). The putative homolog AvrPto*_Pta_*_11528_ shares just 42% sequence identity at the amino acid level. The N- and C-terminal regions are highly conserved but there is significant diversity in the central region required for both the virulence and avirulence functions of this effector in tomato (Chang et al., 2001; Wulf et al., 2004; Xing et al., 2007). Similarly, HopI1*_Pta_*_11528_ shares 55% identity with HopI1*_Pto_*_DC3000_ (Supplementary Figure S6), and both the N- and C-terminal regions are highly conserved maintaining a chloroplast targeting signal and the J domain required for virulence (Jelenska et al., 2007). However, 152 central amino acids containing the proline- and glutamine- (P/Q)-rich repeat region of unknown function are completely absent in HopI1*_Pta_*_11528_. Despite these differences, both the AvrPto and HopI1 homologs in *Pto* DC3000 and *Pta* 11528 strains showed similar abilities to suppress PAMP-induced defense responses, and thus the non-conserved regions may not be required for virulence functions in *N. benthamiana*. The differences between the *Pta* 11528 effectors and the *Pto* DC3000 homologs might indicate functional diversification as a consequence of divergent evolution, as well as diversification to escape recognition.

### Cell Death Promotion and Suppression by *Pto* DC3000 and *Pta* 11528 Effectors

Several *Pto* DC3000 and *Pta* 11528 effectors elicited cell death in *N. benthamiana*. Five effectors including *hopM1_Pto_*_DC3000_, *hopAD1_Pto_*_DC3000_, *hopT1-1_Pta_*_11528_, *avrE1_Pta_*_11528_, and *hopW1*-1*_Pta_*_11528_ strongly elicited necrosis whereas two effectors, *hopAA1-1_Pto_*_DC3000_ and *hopQ1-1_Pto_*_DC3000_ displayed mild chlorosis when expressed transiently in *N. benthamiana* leaves. This supports previous observations indicating that multiple *P. syringae* effectors trigger cell death in *N. benthamiana* (Vinatzer et al., 2006; Wei et al., 2007; Wroblewski et al., 2009; Choi et al., 2017). For example, *hopM1_Pto_*_DC3000_, *hopAA1-1_Pto_*_DC3000_, *hopQ1-1_Pto_*_DC3000_, *avrE1_Pto_*_DC3000_, *hopT1-1_Pto_*_DC3000_, and *hopAD1_Pto_*_DC3000_ effectors elicit cell death in *N. benthamiana* when delivered by *Pseudomonas fluorescens* heterologously expressing a *P. syringae* TTSS (Wei et al., 2007) or though *Pto* DC3000 secretion effector polymutants (Wei et al., 2007, 2015). Similar to our experiments, transient expression of a number of effectors from *Pto* DC3000, *P. syringae* pv. *actinidiae* (*Psa*), and *P. syringae* pv. *syringae* (*Psy*) B728a, showed the ability of *hopQ1-1, avrE1, hopT1-1, hopM1*, and *hopAA1-1* to induce cell death in *N. benthamiana* (Vinatzer et al., 2006; Wroblewski et al., 2009; Choi et al., 2017). It is noteworthy that in our assays, *hopT1-1_PtoDC3000_* did not trigger cell death in *N. benthamiana*, although this has been previously reported (Wei et al., 2007; Wroblewski et al., 2009).

Cell death is also associated with effector recognition by R proteins. This has been reported for HopQ1-1*_Pto_*_DC3000_, and to a much weaker extent for HopAD1*_Pto_*_DC3000_ and the *Pma* ES4326 effector HopW1-1 in *N. benthamiana* (Wei et al., 2007, 2015; Lee et al., 2008). HopQ1-1*_Pto_*_DC3000_ and HopAD1*_Pto_*_DC3000_ are recognized in *N. benthamiana* and consequently, *Pto* DC3000 lacking the *hopQ1-1* or *hopAD1* genes is capable of causing disease symptoms comparable to the virulent *Pta* 11528 (Wei et al., 2007, 2015). The R protein Roq1 mediates recognition of *Xanthomonas* and *Pseudomonas* effector proteins XopQ and HopQ1 in the *Nicotiana* genus (Schultink et al., 2017), whereas in *Arabidopsis*, a genetic interaction between *Qpm3.1* and *hopW1-1* determines resistance to bacterial infection (Luo et al., 2017). However, several lines of evidence suggest that host R proteins do not recognize all effectors that elicited cell death here. Firstly, necrosis induced by *hopM1_Pto_*_DC3000_, *hopAD1_Pto_*_DC3000_, *hopT1-1_Pta_*_11528_, *avrE1_Pta_*_11528_, and *hopW1-1_Pta_*_11528_ effector genes was not dependent on *NbSgt1*, which is typically required for *R* gene function (McDowell and Dangl, 2000). The *NbSgt1*-independence of *avrE1* induced cell death has been reported previously (Choi et al., 2017). However, cell death induced by the *Psy* B728a effector *hopM1* and the *Psa* effectors *hopT1-1* and *hopW1-1* were reported to be at least partially dependent on *NbSgt1* (Vinatzer et al., 2006; Choi et al., 2017). Therefore, further analyses are required to determine to what extent the cell death caused by these effectors is dependent on *NbSgt1* in *N. benthamiana.* On the other hand, *NbSgt1* may not be required for all *R* gene-mediated plant defenses and thus, correlation of *NbSgt1*-dependency with effector recognition by R proteins should be undertaken cautiously. Secondly, *Pta* 11528 is virulent on *N. benthamiana* despite possessing a HopW1-1 homolog. It is possible that some of these effectors would not elicit cell death when expressed under native conditions, i.e., in bacteria and delivered to the plant cell via the TTSS. And thirdly, there is precedence for host cell death to be associated with effector virulence, as demonstrated for members of the AvrE1 and HopM1 effector families (Badel et al., 2006; Boureau et al., 2006; Ham et al., 2006). Both *avrE1* and *hopM1* are functionally redundant and reside in the conserved effector locus (CEL) of the *P. syringae* Hrp pathogenicity island (DebRoy et al., 2004; Badel et al., 2006; Kvitko et al., 2009). HopM1 and AvrE1 are required to establish a water-soaked aqueous living space in plants that is crucial for virulence (Xin et al., 2016). Whether HopAA1-1, another effector encoded in the CEL locus, or the remaining cell death-inducing effectors of *Pto* DC3000 and *Pta* 11528 function similarly to HopM1/AvrE in altering apoplastic environmental conditions is unknown. The conservation of necrosis-inducing effectors in various bacteria suggests this function may be an important virulence strategy for bacterial infections on plants.

A number of *Pto* DC3000 and *Pta* 11528 effectors masked the activity of cell death-inducing effectors when co-expressed transiently in *N. benthamiana*. Similar data exist for suppression of effector-triggered cell death phenotypes by *Pto* DC3000 and *Psa* effectors (Jamir et al., 2004; Guo et al., 2009; Wei et al., 2015; Choi et al., 2017). Indeed, most *Pto* DC3000 effectors that acted as cell death suppressors in this work where previously identified as suppressors of the HR response induced by HopA1 in tobacco when delivered by *Pseudomonas fluorescens* heterologously expressing a *P. syringae* TTSS (Guo et al., 2009). Notably, it has been reported recently that HopQ1-mediated cell death suppression in *N. benthamiana* is due to attenuation of *Agrobacterium*-mediated protein expression rather than a genuine virulence activity. Thus, it is possible that additional effectors may act in a similar fashion (Adlung and Bonas, 2017). We speculate that necrosis induction by effectors must be tightly regulated to establish and maintain an optimal intracellular niche. Although it is possible that some effectors would not interfere with cell death when expressed at native levels, the potent activity of some of them suggests that cell death suppression may occur during infection to at least some extent. On the other hand, the ability of some effectors to interfere with the outcomes of others identifies which effectors perturbate common defensive pathways. HopAD1*_Pto_*_DC3000_-, HopM1*_Pto_*_DC3000_-, and HopT1-1*_Pta_*_11528_-dependent cell death could be efficiently suppressed by multiple effectors from both *Pto* DC3000 and *Pta* 11528 repertoires, which include both AvrPtoB homologs. This supports recent work by Wei et al. (2015) indicating interplay between HopAD1 and AvrPtoB in regulating HopAD1-dependent cell death. Despite this, *Pto* DC3000 effectors could not collectively suppress the HR events activated upon recognition of AvrPtoB and/or AvrPto in transgenic *N. benthamiana* plants expressing the tomato *Pto* and *Prf* resistance genes (Balmuth and Rathjen, 2007). In addition, neither the effector complements of *Pto* DC3000 or *Pta* 11528 were able to collectively suppress HopQ1-1 or HopAD1 induced defenses in *N. benthamiana* (Wei et al., 2007, 2015). Interplay between effectors within the bacterial repertoires is emerging as an important but poorly understood phenomenon controlling defensive phenotypic outcomes (Wei et al., 2015; Huett, 2017). More studies are needed in this area to understand the complex network of interactions between the effectors among the repertoires and how this translates into an effective immune response or a virulence strategy to promote disease.

### *Pseudomonas syringae* Effectors Differentially Suppress Innate Immune Responses

In recent years, many effectors have been shown to suppress PAMP-induced defenses (Macho and Zipfel, 2015; Buttner, 2016; Toruno et al., 2016). Here, we analyzed the ability of effectors to interfere with PAMP-triggered defenses activated between seconds to minutes to hours after elicitation, which has allowed us to group effectors into three major categories based on their range of suppressive activities.

Group A effectors acted as broad-range suppressors of all early PAMP-induced responses tested. AvrPto*_Pto_*_DC3000_ and AvrPtoB*_Pto_*_DC3000_ which fall into this group, are well known suppressors of flg22-induced defenses by targeting PRR complexes directly at the plasma membrane (Chinchilla et al., 2007; Heese et al., 2007; Gohre et al., 2008; Xiang et al., 2008). The additional *Pto* DC3000 and *Pta* 11528 effectors within Group A may also target PRR complexes due to their comprehensive suppression activities. Indeed, direct inactivation or destabilization of PAMP receptor complexes at the plasma membrane is a common strategy of multiple effectors (Macho and Zipfel, 2015; Buttner, 2016; Toruno et al., 2016). HopM1*_Pto_*_DC3000_ suppresses vesicle trafficking to overcome host innate immunity (Nomura et al., 2006). Our data indicates additional roles for this effector in early PAMP signaling suppression. Both HopM1*_Pto_*_DC3000_ and HopQ1-1*_Pto_*_DC3000_ associate with multiple 14-3-3 proteins (Nomura et al., 2006; Giska et al., 2013; Li et al., 2013; Lozano-Duran et al., 2014). 14-3-3 proteins are highly conserved eukaryotic regulatory adapters whose interaction with client proteins can regulate protein activity and remarkably, chemical inhibition of 14-3-3s results in suppression of the PAMP-triggered ROS burst (Lozano-Duran et al., 2014). Some 14-3-3 proteins associate with several defense-related proteins *in planta*, including the FLS2 co-receptor BAK1 (Chang et al., 2009). Whether HopM1*_Pto_*_DC3000_ and HopQ1-1*_Pto_*_DC3000_ act through interaction with 14-3-3s remains to be explored.

Group B effectors suppressed the PAMP-induced ROS burst not the Ca^2+^ influx. These included HopT1-1*_Pto_*_DC3000_, HopX1*_Pta_*_11528_, and HopAR1*_Pta_*_11528._ These effectors abolished the PAMP-induced ROS burst with differential impact on activation of defense gene markers. While HopT1-1*_Pto_*_DC3000_ compromised flg22- and chitin-dependent induction of defense genes, HopX1*_Pta_*_11528_ and HopAR1*_Pta_*_11528_ did not. Furthermore, their ROS-suppressive action was restricted to chitin-induced responses. The *P. syringae* pv. *phaseolicola* effector HopAR1 suppresses PTI by directly targeting BIK1 and other RLCKs (Zhang et al., 2010), which contrast with our results. However, the *hopAR1_Pta_*_11528_ and *hopAR1_Pph_*_race3_ versions of this effector differ significantly in their protein sequences. HopAR1*_Pta_*_11528_ contains an extra 44 N-terminal amino acids (in a relatively small effector of 311 amino acids), whereas the common C-terminal domain shares a moderate 78% identity, which may account for the observed differences. In an alternative model, HopX1*_Pta_*_11528_ targets conserved JAZ transcriptional repressors to activate JA signaling and promote infection in *Arabidopsis* (Gimenez-Ibanez et al., 2014), and therefore, additional targets would be expected for this effector. This is unsurprising as increasing evidence suggests that effector proteins target multiple host proteins simultaneously in a “death by a thousand cuts” strategy to abolish defense responses (Navarro et al., 2008; Shan et al., 2008; Gimenez-Ibanez et al., 2009).

Group C effectors are suppressors of PAMP-induced gene induction. This group only compromised PAMP-induced gene expression but not the other outputs. They include the *Pto* DC3000 effectors HopF2*_Pto_*_DC3000_, HopAF1*_Pto_*_DC3000_, HopI1*_Pto_*_DC3000_, and HopH1*_Pto_*_DC3000_, and the *Pta* 11528 effectors HopF1*_Pta_*_11528_ and HopI1*_Pta_*_11528_. Because this group suppressed gene induction but not plasma membrane-related events, we suggest that they act later in signal transduction perhaps proximal to or within the nucleus. In this group falls HopF2*_Pto_*_DC3000_, which was previously shown to suppress *Arabidopsis* immunity by targeting the co-receptor BAK1 (Zhou et al., 2014). This finding is difficult to reconcile with our data as this effector did not suppress early flg22-induced Ca^2+^ and ROS bursts in our experiments to any extent. This is similar to HopB1*_Pto_*_DC3000_, one of the few effectors that did not suppress any of the defensive outputs tested in this work, despite the fact that it acts as a protease that targets BAK1 (Li et al., 2016). On the other hand, HopAF1*_Pto_*_DC3000_ suppresses ethylene production (Washington et al., 2016) whereas HopI1 targets plant heat shock chaperone protein Hsp70 (Jelenska et al., 2010). None of these functions explains the ability of these effectors to suppress PAMP-induced gene expression. Thus, we expect alternative targets for these effectors to disrupt transcriptional activation. Effectors that interfere with the transcriptional machinery are not yet known from *Pto* DC3000, but are well described in other pathogens such as *Xanthomonas* which produces transcription activator-like effectors (TALEs) (Boch et al., 2009; Moscou and Bogdanove, 2009). Whether *Pto* DC3000 also injects direct modulators of host gene expression remains an open question for the future.

### Most Effectors Interfere With Responses Elicited by Different PAMPs

Most effectors interfered with both flg22- and chitin-induced defense responses simultaneously, indicating that a considerable overlap exists between these pathways. One exception was the AvrPto homologs of both *Pto* DC3000 and *Pta* 11528 which suppressed flagellin signaling but not chitin responses. This was surprising because AvrPto*_Pto_*_DC3000_ was previously reported to block chitin-induced defense gene expression in transgenic *Arabidopsis* protoplasts (Shan et al., 2008). It is possible that the chitin-suppressing effect of AvrPto*_Pto_*_DC3000_ was simply due to overexpression in *Arabidopsis*. Alternatively, the kinase domain of the *Arabidopsis* chitin receptor/receptor complex might be simply a better target for AvrPto*_Pto_*_DC3000_ than its *N. benthamiana* homolog (Shan et al., 2008; Xiang et al., 2008). Therefore, it remains to be determined how chitin-induced signaling in *N. benthamiana* evades AvrPto-mediated suppression. Conversely, HopX1*_Pta_*_11528_ and HopAR1*_Pta_*_11528_ suppressed chitin-induced ROS generation, but not that generated by flg22 treatment. This might be simply a consequence of the different strength of ROS levels induced by both elicitors. While flg22 induces a very strong response, the chitin response is relatively mild. Therefore, smaller effects might be more easily revealed using chitin as an elicitor. Overall, much of our data suggest that chitin- and flg22-induced signaling converge soon after PAMP perception, since most effectors suppressed defense responses induced by both elicitors.

### Final Considerations

*Nicotiana benthamiana* has emerged as an important and widely used experimental system to study plant biology. Its advantages include its large leaves, its high amenability for *A. tumefaciens*-mediated transient expression, the possibility to rapidly test the involvement of host factors using VIGS, and the reproducibility of the data (77, 78). *Agrobacterium*-mediated transient expression has already been used to study some effectors that suppress plant defense responses at the molecular level (24, 79). Thus, the efficiency and versatility of the agroinfiltration technique in *N. benthamiana* prompted us to investigate the ability of a large number of *P. syringae* type III effectors to suppress a collection of defense responses activated upon PAMP treatment. However, it should be taken into account that overexpression of effector proteins can led to misleading results by causing protein imbalances, promiscuous interactions, and regulation of pathways that are associated with the degree of overexpression rather than the function of the protein. On the other hand, overexpression of effector proteins in plant cells, combined with loss-of-function analyses, is a common and powerful approach to understanding effector function. Identification of effector targets is the next step to unraveling how pathogens can overcome plant immunity and promote pathogenesis. This will lead ultimately to new strategies for crop protection in the field.

## Author Contributions

DH, JR, and SG-I designed the experiments. DH, CS, and SG-I conducted the experiments. DH, JR, and SG-I analyzed the data. JR, JC, TB, and SG-I wrote the manuscript. All authors edited and approved the final manuscript.

## Conflict of Interest Statement

The authors declare that the research was conducted in the absence of any commercial or financial relationships that could be construed as a potential conflict of interest. The reviewer BK declared a past co-authorship with one of the authors JC to the handling Editor.
