# Supplementary material for: Differential Suppression of Nicotiana benthamiana Innate Immune Responses by Transiently Expressed Pseudomonas syringae Type III Effectors
Source: Front Plant Sci. 2018 May 23;9:688. doi: 10.3389/fpls.2018.00688 (PMC5974120; doi:10.3389/fpls.2018.00688)
Supplement: Supplementary file 1 [file Presentation_1.pdf]

Supplementary Material

Differential suppression of *Nicotiana benthamiana* innate immune responses by transiently expressed *Pseudomonas syringae* type III effectors

Selena Gimenez-Ibanez[1,2†], Dagmar R. Hann[1,3,4†], Jeff H. Chang[5, 6], Cécile Segonzac[1,7], Thomas Boller[3] and John P. Rathjen[1,8]

[1] The Sainsbury Laboratory, Colney, Norwich, NR4 7UH, UK.

[2] Plant Molecular Genetics Department, Centro Nacional de Biotecnología-CSIC (CNB-CSIC), Madrid, 28049, Spain.

[3] Department of Environmental Science, Botanical Institute, University of Basel, Hebelstrasse 1, 4051 Basel, Switzerland.

[4] Institute of Genetics, Ludwig-Maximilians-Universität, Munich (LMU), Grosshaderner Str. 2-4, D-82152 Martinsried, Germany.

[5] Department of Botany and Plant Pathology, 4, Oregon State University, Corvallis, OR 97331, USA.

[6] Center for Genome Research and Biocomputing, Oregon State University, Corvallis, OR 97331, USA.

[7] Department of Plant Science, Plant Genomics and Breeding Institute and Research Institute of Agriculture and Life Sciences, College of Agriculture and Life Sciences, Seoul National University Seoul, Seoul 151-921, South Korea.

[8] Research School of Biology, The Australian National University, 134 Linnaeus Way, Acton 2601, ACT, Australia.

[†]These authors contributed equally to this work.

*****To whom correspondence should be addressed. E-mail: selena.gimenez@cnb.csic.es

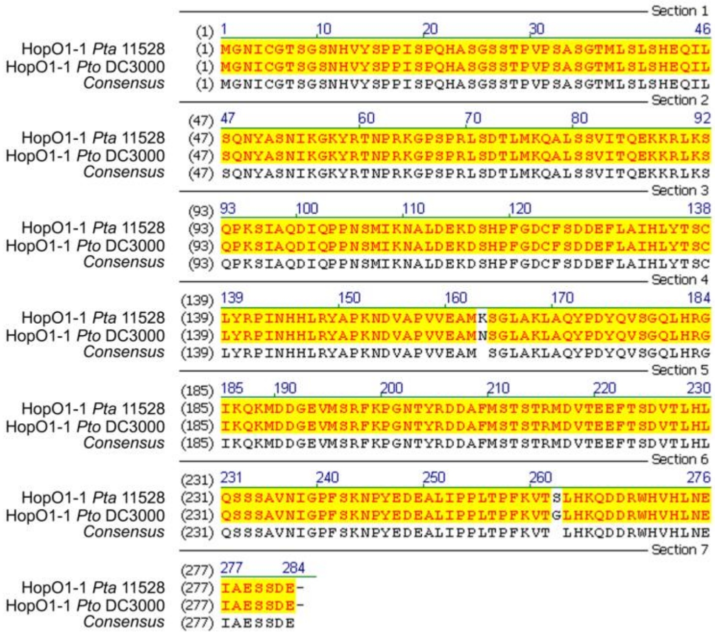

Figure S1. Sequence alignment of HopO1-1$_{\textit{Pta}11528}$.

Amino acid alignment of HopO1-1$_{\textit{Pta}11528}$ and HopO1-1$_{\textit{Pto}\mathrm{DC}3000}$. Identical residues are labelled in yellow.

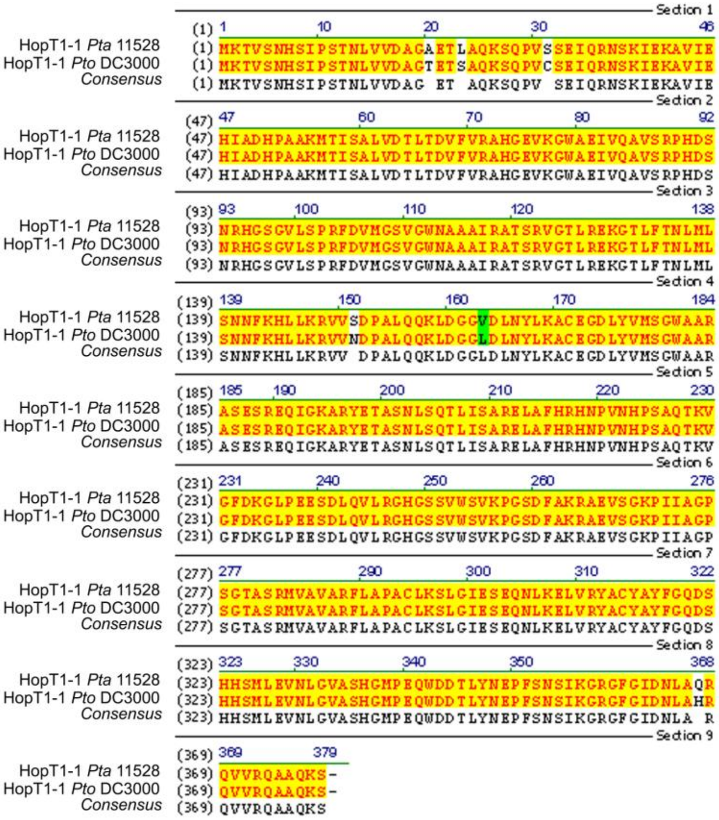

Figure S2. Sequence alignment of HopT1-1*Pta*11528.

Amino acid alignment of HopT1-1*Pta*11528 and HopT1-1*Pto*DC3000. Identical residues are labelled in yellow and conservative substitutions in green.

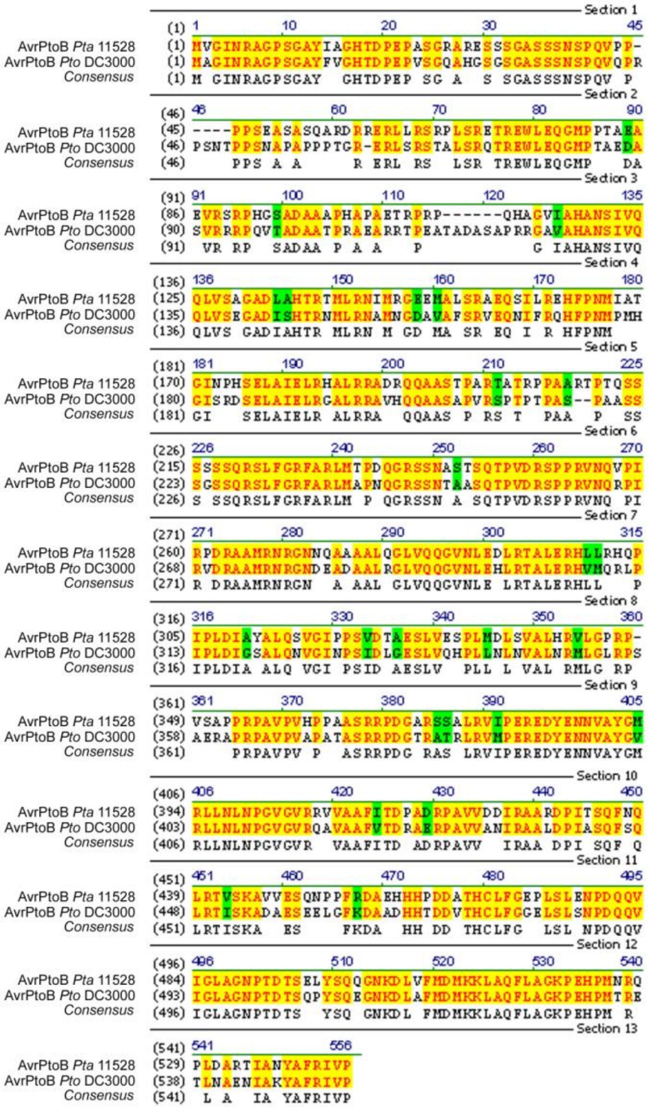

Figure S3. Sequence alignment of AvrPtoB$_{Pta11528}$.

Amino acid alignment of AvrPtoB$_{Pta11528}$ and AvrPtoB$_{PtoDC3000}$. Identical residues are labelled in yellow and conservative substitutions in green.

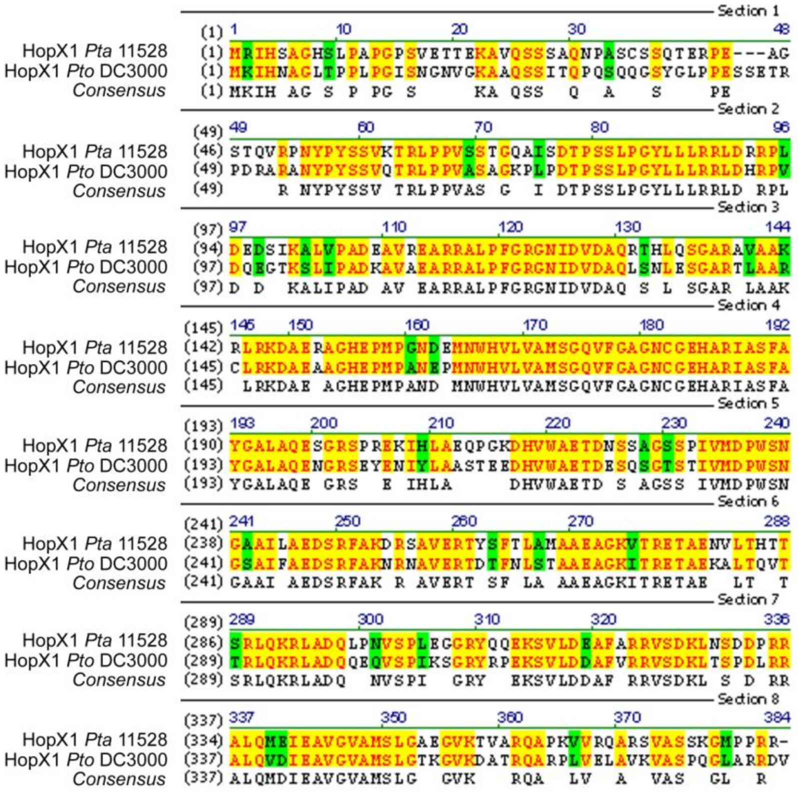

Figure S4. Sequence alignment of HopX1$_{Pta11528}$.

Amino acid alignment of HopX1$_{Pta11528}$ and HopX1$_{PtoDC3000}$. Identical residues are labelled in yellow and conservative substitutions in green.

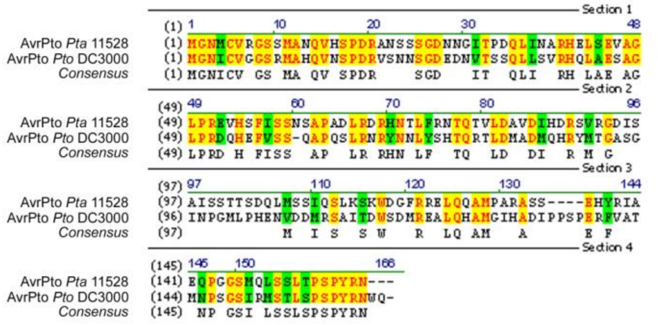

Figure S5. Sequence alignment of AvrPto*Pta*11528.

Amino acid alignment of AvrPto*Pta*11528 and AvrPto*Pto*DC3000. Identical residues are labelled in yellow and conservative substitutions in green.

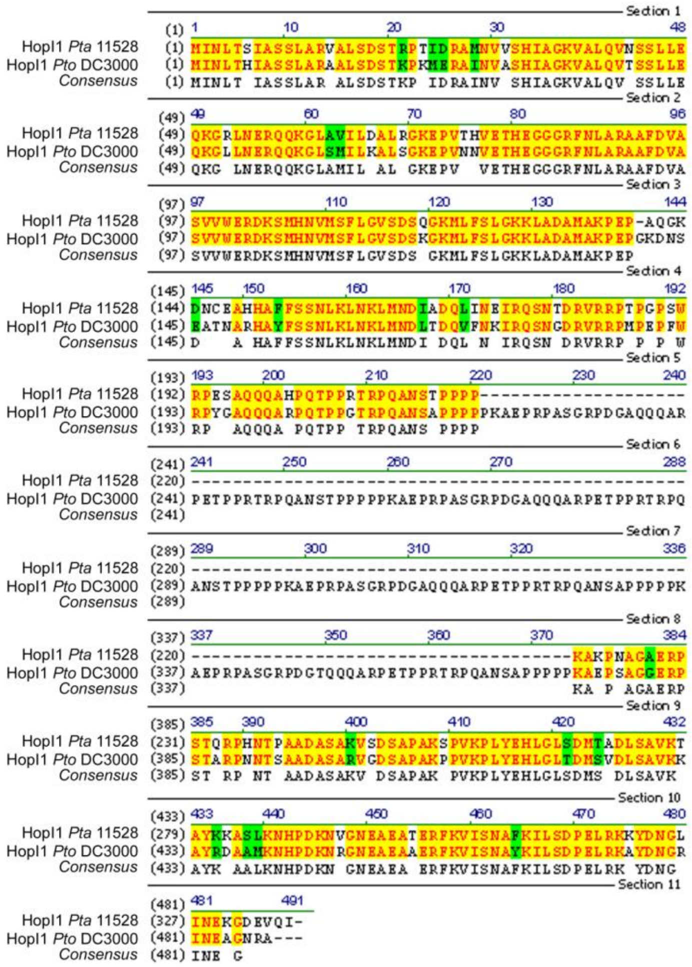

Figure S6. Sequence alignment of HopI1$_{Pta11528}$.

Amino acid alignment of HopI1$_{Pta11528}$ and HopI1$_{PtoDC3000}$. Identical residues are labelled in yellow and conservative substitutions in green.

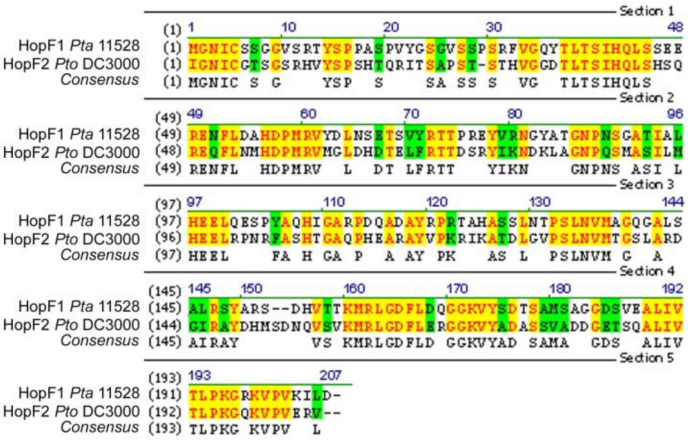

Figure S7. Sequence alignment of HopF1*Pta*11528.

Amino acid alignment of HopF1*Pta*11528 and HopF2*Pto*DC3000. Identical residues are labelled in yellow and conservative substitutions in green.

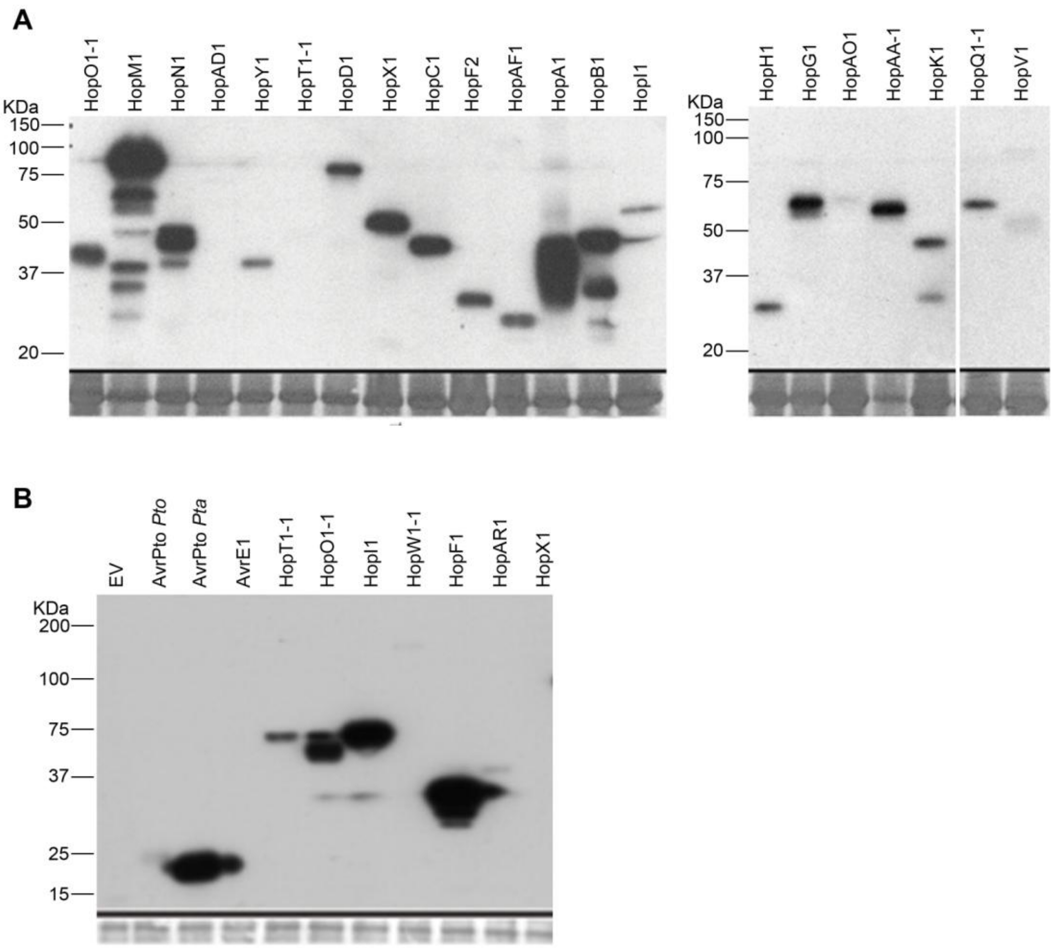

Figure S8. Accumulation of *Pto* DC3000 and *Pta* 11528 effector proteins in *N. benthamiana*.

(A) Western blot showing accumulation of *Pto* DC3000 effector proteins in leaf tissue after *A. tumefaciens* mediated transient expression in *N. benthamiana* for two days. A Coomassie brilliant blue loading control is shown in the lower panel.

(B) Western blot showing accumulation of all *Pta* 11528 effector proteins in leaf tissue after *A. tumefaciens* mediated transient expression in *N. benthamiana* for two days. A Coomassie brilliant blue loading control is shown in the lower panel.

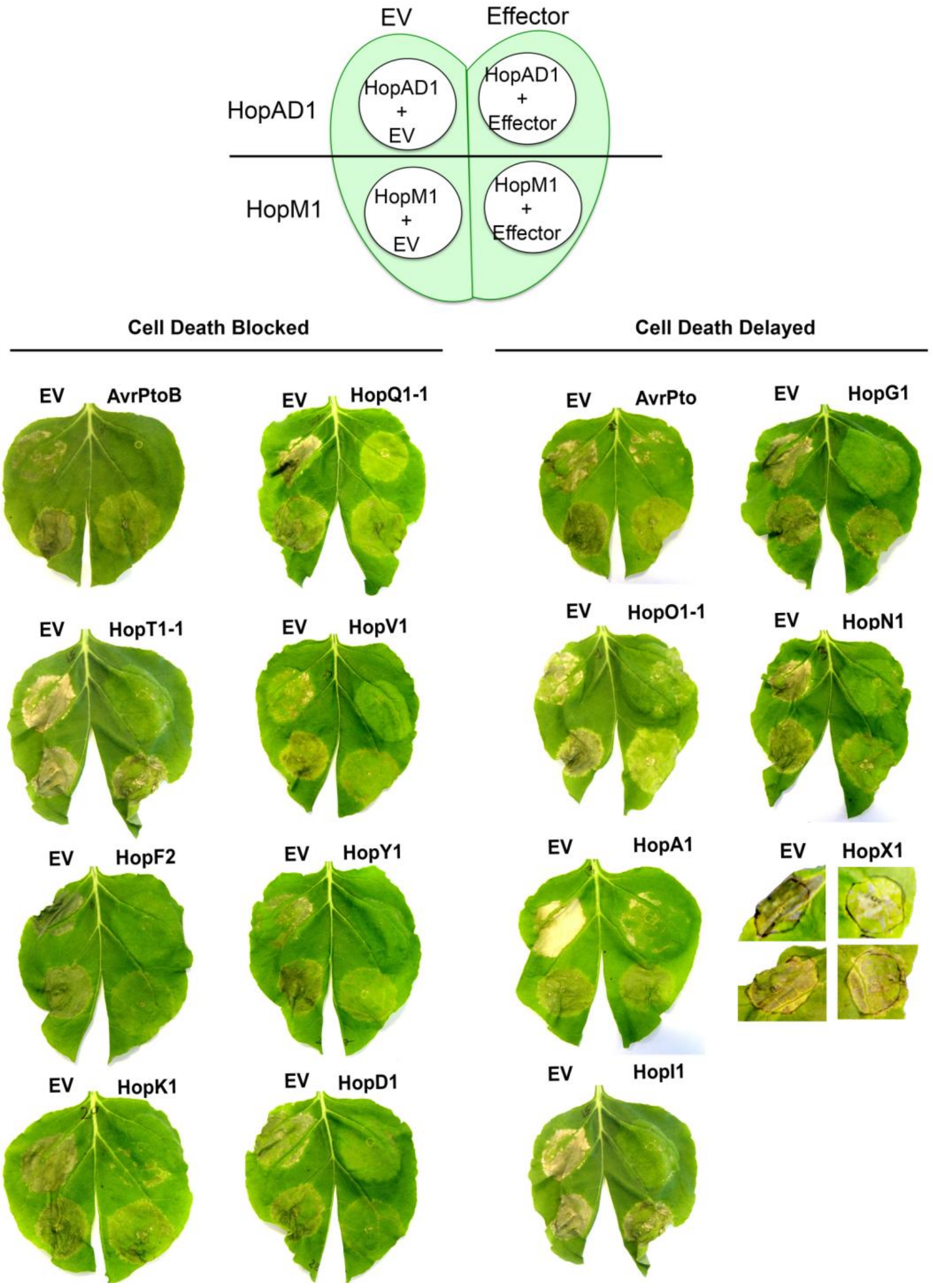

Figure S9. Most *Pto* DC3000 effectors can interfere with $hopM1_{Pto\mathrm{DC3000}}$-induced cell death.

Suppression of $hopM1_{Pto\mathrm{DC3000}}$ and $hopAD1_{Pto\mathrm{DC3000}}$ induced cell death by *Pto* DC3000 effectors. The effector $hopM1_{Pto\mathrm{DC3000}}$ or $hopAD1_{Pto\mathrm{DC3000}}$ was co-expressed in *N. benthamiana* leaves with each effector in the *Pto* DC3000 repertoire, or an *EV* control, as depicted in the upper schematic diagram. Each of the two *A. tumefaciens* strains containing the respective effector gene or an EV control were prepared at OD_{600}=0.5 then mixed in equal volumes. Cell death induced by the effectors was scored 6 days after infiltration. Similar results were obtained in two independent experiments.

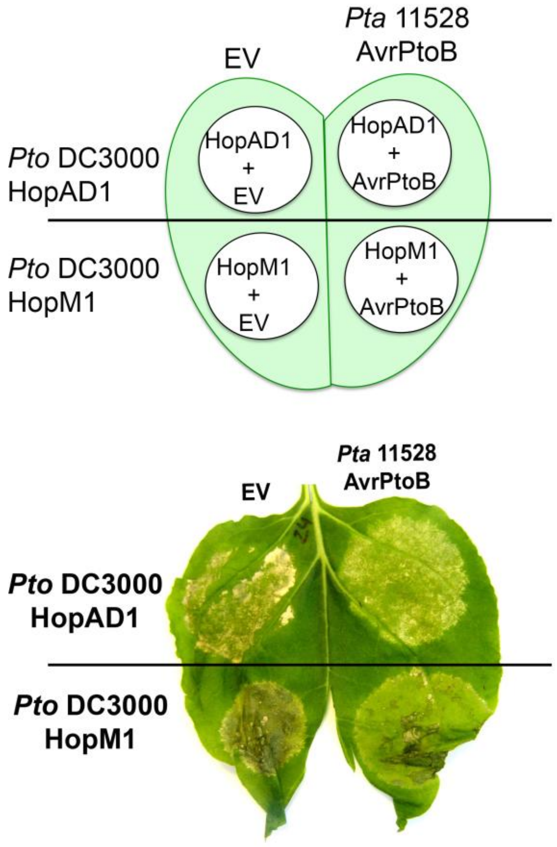

Figure S10. *Pta* 11528 AvrPtoB can interfere with *hopAD1*$_{Pto\mathrm{DC3000}}$ or *hopM1*$_{Pto\mathrm{DC3000}}$-induced cell death.

Suppression of *hopM1*$_{Pto\mathrm{DC3000}}$ and *hopAD1*$_{Pto\mathrm{DC3000}}$ induced cell death by the *Pta* 11528 effector AvrPtoB. The effector *hopM1*$_{Pto\mathrm{DC3000}}$ or *hopAD1*$_{Pto\mathrm{DC3000}}$ was co-expressed in *N. benthamiana* leaves with *avrPtoB*$_{Pta\mathrm{11528}}$ or an *EV* control, as depicted in the upper schematic diagram. Each of the two *A. tumefaciens* strains containing the respective effector gene or an EV control were prepared at OD_{600}=0.5 then mixed in equal volumes. Cell death induced by the effectors was scored 6 days after infiltration. Similar results were obtained in two independent experiments.

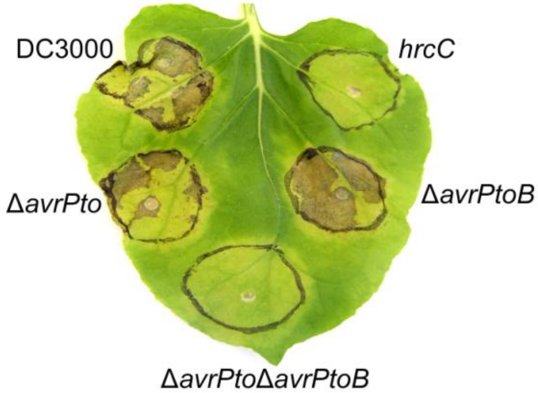

Figure S11. *Pto* DC3000 effectors cannot collectively suppress the induction of the hypersensitive response (HR) triggered upon recognition of AvrPto$_{\textit{Pto}\text{DC3000}}$ and/or AvrPtoB$_{\textit{Pto}\text{DC3000}}$ in transgenic *N. benthamiana* plants expressing the tomato *Pto* and *Prf* genes (*N. benthamiana* R411A).

Transgenic *N. benthamiana* plants were infiltrated with a high dose ($5x10^7$ cfu/ml) of *Pto* DC3000 (DC3000), *Pto* DC3000 *hrcC* (*hrcC*), *Pto* DC3000Δ*avrPto* (Δ*avrPto*), *Pto* DC3000Δ*avrPtoB* (Δ*avrPtoB*) and *Pto* DC3000Δ*avrPto*Δ*avrPtoB* (Δ*avrPto* Δ*avrPtoB*). The HR was visible 24-48 h after infiltration, and photographed at 3 days post infiltration. Similar results were obtained in two independent experiments.
